# Austalides S-U, New Meroterpenoids from the Sponge-Derived Fungus *Aspergillus aureolatus* HDN14-107

**DOI:** 10.3390/md14070131

**Published:** 2016-07-14

**Authors:** Jixing Peng, Xiaomin Zhang, Wei Wang, Tianjiao Zhu, Qianqun Gu, Dehai Li

**Affiliations:** 1 Key Laboratory of Marine Drugs, Chinese Ministry of Education, School of Medicine and Pharmacy, Ocean University of China, Qingdao 266003, China; pengjixing1987@163.com (J.P.); xiaominzhang91@163.com (X.Z.); wwwakin@ouc.edu.cn (W.W.); zhutj@ouc.edu.cn (T.Z.); guqianq@ouc.edu.cn (Q.G.); 2 Laboratory for Marin Drugs and Bioproducts, Qingdao National Laboratory for Marine Science and Technology, Qingdao 266237, China

**Keywords:** austalide, fungus, influenza virus A

## Abstract

Three new meroterpenoids, named austalides S-U (**1**–**3**), were isolated from the culture of a sponge-derived fungus *Aspergillus aureolatus* HDN14-107, together with eleven known austalides derivates (**4**–**14**). Their structures, including absolute configurations, were assigned on the basis of NMR, MS data, and TDDFT ECD calculations. Compound **1** is the first case of austalides with the terpene ring fused to the chroman ring in *trans* configuration. Compounds **3** and **5** exhibited activities against influenza virus A (H1N1), with IC_50_ values of 90 and 99 μM, respectively.

## 1. Introduction

The austalides are a family of meroterpenoid produced mainly by marine-derived *Aspergillus* and *Penicullium* genera, 28 members have been reported since the first one discovered in 1984 [1,2,3,4,5]. Biosynthetically, they are derived from 6-[(2*E*,6*E*)farnesyl]-5,7-dihydroxy-4-methylphthalide [5], followed by cyclization and oxidative modification. Austalides possess diverse structures, which could be divided into four subfamilies corresponding to ring systems including 5/6/6/6 tetracyclic, 5/6/6/6/6 pentacyclic, 5/6/6/6/7 pentacyclic and 5/6/6/6/5/6 hexacyclic rings. Among the austalide meroterpenoids, those containing 5/6/6/6/6 pentacyclic ring system are rare, with only three cases reported including austalides K and L [2], and 17*S*-dihydroaustalide K [5]. In addition, the terpene rings are fused to the chroman ring in *cis* configuration with 11*S*, 21*R* for most of the austalides. Although the structures are attractive, only limited bioactivities for few of them have been reported such as antibacterial and *endo*-1,3-β-d-glucanase inhibitory activities [4,5].

**Figure 1:**
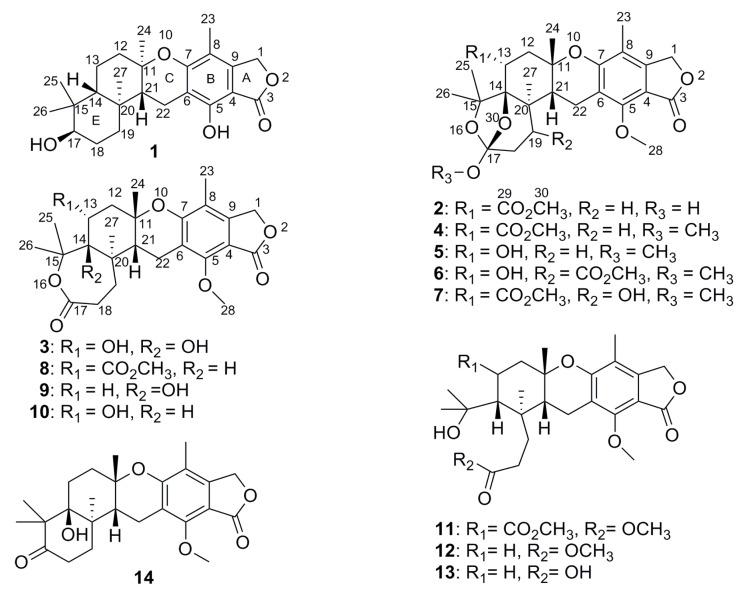
Structures of compounds **1**–**14**.

As part of our continuing efforts to discover bioactive products from sponge-derived microorganisms [6,7,8,9,10,11,12], an *Aspergillus aureolatus* strain HDN14-107, isolated from an unidentified sponge collected at Xisha Island, China, was investigated which resulted in the discovery of three new meroterpenoids, named austalides S-U (**1**–**3**), together with eleven known ones (**4**–**14**) (Figure 1). The structures of new compounds were identified by NMR and HRESIMS, and the absolute configurations were determined by comparison of the experimental ECD spectra as well as the time-dependent density functional theory electronic circular dichroism (TDDFT ECD) calculations. Among them, compound **1** is the first austalide with the terpene ring fused to the chroman ring in *trans* configuration and also is the fourth case of analogues with 5/6/6/6/6 pentacyclic ring system. Compounds **3** and **5** exhibited anti-influenza virus A (H1N1) activity with IC_50_ values of 90 and 99 μM, respectively. Herein, we report the isolation, structure elucidation and bioactivities of the new compounds.

## 2. Results and Discussion

**Figure 2:**
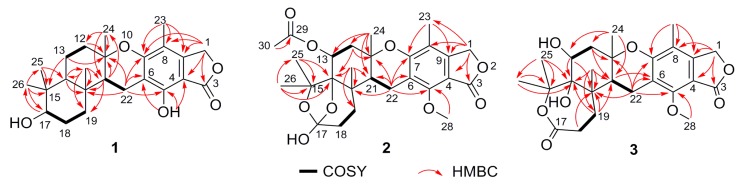
Key COSY and HMBC correlations of **1**–**3**.

The molecular formula of austalide S (**1**) was determined as C_24_H_32_O_5_ according to the protonated HRESIMS peak at *m*/*z* 401.2323 (Supplementary Figure S1). The ^1^H and ^13^C NMR (Table 1 and Table 2) spectra indicated the presence of five methyls, appearing as singlet in the ^1^H NMR spectrum, including one aromatic methyl group at *δ*_H_ 2.05 ppm (CH_3_-23), and four aliphatic ones at *δ*_H_ 1.34, 0.99, 0.96 and 0.78 ppm. Additionally, six methylenes with one oxygenated one at *δ*_H_ 5.12 (C-1), three methines, and ten non-protonated carbons were observed. A comparison of the 1D NMR data (Table 1 and Table 2) with those for 17*S*-dihydroaustalide K [5] showed high similarity. The key differences were the replacement of the methoxy group in 17*S*-dihydroaustalide K by a hydroxy group (5-OH) in **1**, which was further confirmed by the COSY and HMBC correlations (Figure 2). Although the planar structures of **1** and 17*S*-dihydroaustalide K are highly similar, the chemical shifts of C-11, C-12 and C-24 are significantly different (*δ*_C_ 77.2, 43.6 and 24.7 in **1** vs. 76.5, 40.4 and 27.3 in 17*S*-dihydroaustalide K, respectively), indicating that their stereochemistry might be different.

**Figure 3:**
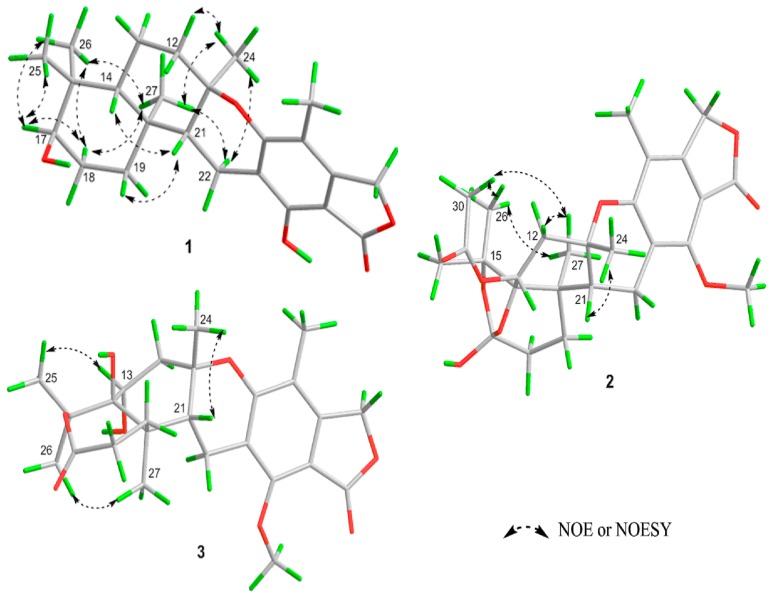
Key NOESY and NOE correlations of **1**–**3**.

The relative configuration of **1** was deduced from analysis of the NOESY and NOE correlations (Figure 3). The NOEs observed between CH_3_-27 and CH_3_-24 indicated that they faced to the same side of ring D. The NOESY correlations of H-14/H-21/H-19 suggested that they oriented to the opposite side of ring D. Due to the chair conformation of ring E, the position of 17-OH group was deduced as axial and on the same face with H-14 based on the NOESY correlations between H-17 and H-25/H-26 which indicated the equatorial position of H-17. Thus, the relative stereochemistry of **1** was established as (11R*, 14R*, 17R*, 20S*, 21R*).

**Figure 4:**
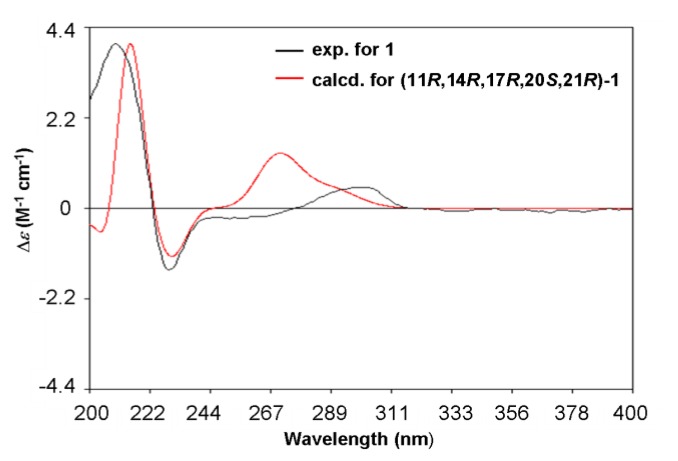
B3LYP/6-31+G(d)-calculated ECD spectra of (11*R*, 14*R*, 17*R*, 20*S*, 21*R*)-**1** (**red**), and the experimental ECD spectrum of **1** (**black**). (σ = 0.20 eV).

Given the ECD have been provided to be a straightforward way for the configurational assignment of austalides [3], the TDDFT ECD (time-dependent density functional theory electronic circular dichroism) calculations of alternative solution conformers of **1** were carried out. The calculated ECD curves (Figure 4) of the (11*R*, 14*R*, 17*R*, 20*S*, 21*R*)-**1** gave a good agreement with the experimental data of compound **1**, indicating that the absolute configuration of **1** was 11*R*, 14*R*, 17*R*, 20*S*, 21*R*. It is the first case for austalides with 11*R* configuration.

**Figure 5:**
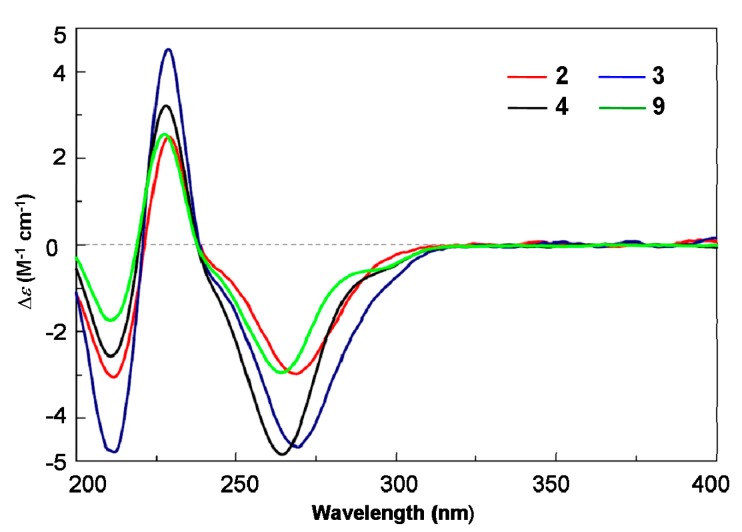
Experimental ECD spectra of compounds **2**, **3**, **4** and **9**.

Austalide T (**2**) was obtained with the molecular formula C_27_H_34_O_9_ based on the protonated peak at *m*/*z* 503.2276 (Supplementary Figure S10). The 1D NMR data of **2** were almost identical to those of the known austalide A (**4**), except for the disappearance of the methoxyl at C-17 in **4**, suggesting that **2** possesses the same skeleton as **4** but with a 17-OH group, which agreed with the 14 amu molecular weight loss. Accordingly, slight upfield shifts were observed for C-17 (*δ*_C_ = 117.8 ppm) in the ^13^C NMR spectra of **2**, compared to C-17 (*δ*_C_ = 119.3 ppm) of **4**. The planar structure of **2** was also confirmed by COSY and HMBC correlations (Figure 2). Moreover, the NOE spectrum indicated a (11S*, 13R*, 14R*, 17S*, 20R*, 21R*) relative configuration, in agreement with **4**. The absolute configuration of **2** was also inferred to be the same as **4** based on the similarity of their ECD spectra (Figure 5), which allowed us to determine the absolute configuration of **2** as 11*S*, 13*R*, 14*R*, 17*S*, 20*R*, 21*R*.

The HRESIMS of austalide T (**3**) indicated the molecular formula C_25_H_32_O_8_ (Supplementary Figure S18), with one more oxygen atoms than austalide J (**9**). Their ^1^H and ^13^C NMR data (Table 1 and Table 2) revealed that **3** and **9** shared the same pentacyclic skeleton. The differences were attributed to the appearance of a C-14 hydroxy group in **9**, and two hydroxy groups in **3** which were located at C-13 and C-14, respectively, according to the chemical shifts (*δ*_C_ 69.1, C-13 and *δ*_C_ 86.6, C-14 in **3**), and the COSY and HMBC correlations (Figure 2). The relative configuration of **3** was also determined as (11S*, 13R*, 14R*, 20R*, 21R*) by interpretation of the NOE correlations. The absolute configuration was further established to be 11*S*, 13*R*, 14*R*, 20*R*, 21*R* based on the Cotton effects at 270 nm (Δε −4.65), 229 nm (Δε +4.53) and 212 nm (Δε −4.78) which were similar to those of **9** (Figure 5).

Those known compounds **4**–**14** were identified as austalides A, B, D, E, G, I, J, L, P (**4**–**7**, **11**, **8**–**9**, **12**, **14**) [1,2,3,4], 13-*O*-deacetyaustalide I (**10**) [5] and austalide P acid (**13**) [5], respectively, by comparison of their spectroscopic and physical data (^1^H and ^13^C NMR, MS, and ECD) with those reported in the literature.

All the compounds showed no cytotoxic activity (IC_50_ > 50 μM). The ant-influenza A virus (H1N1) activities of compounds **1**–**14** were evaluated by the CPE inhibition assay [8,13]. Compounds **3**, **5**, **8** and **13** showed inhibitory effects with IC_50_ values of 90, 99, 131 and 145 μM, respectively, while other compounds were inactive (IC_50_ > 200 μM) (ribavirin as positive control, IC_50_ 102 μM).

**Scheme 1:**
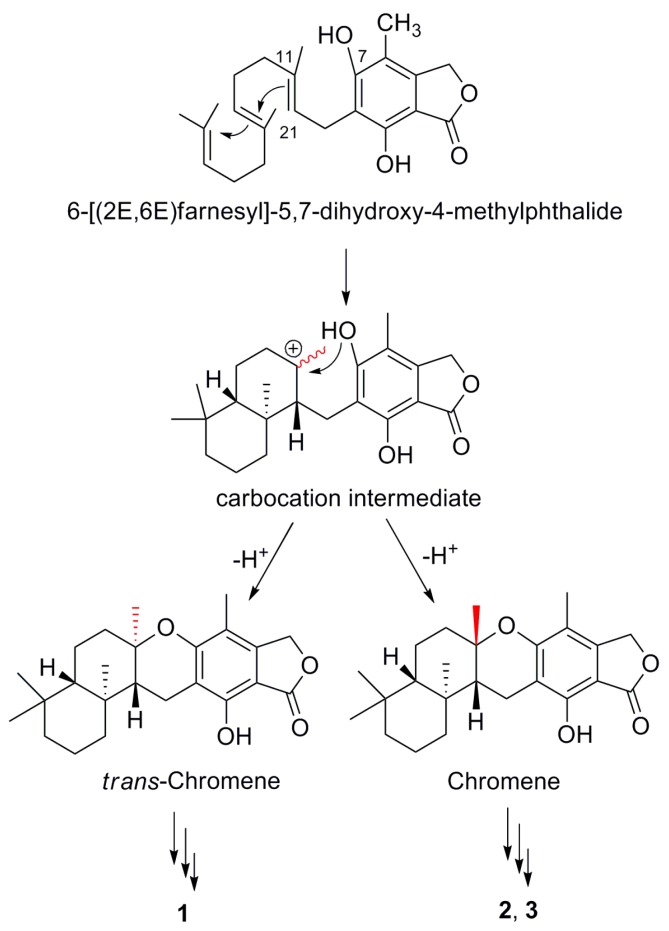
A possible biosynthetic route to **1**–**3**.

Previous biosynthetic studies have revealed a hybrid polyketide pathway and mevalonate pathway for austalides [1,2,3,4,5]. By forming an intermediate with carbocation [14,15], compounds **1**–**3** were proposed to be generated through a pair of epimers with chromene and *trans*-chromene moieties (Scheme 1), following with further modifications.

## 3. Experimental Section

### 3.1. General Experimental Procedures

Optical rotations were measured with a JASCO P-1020 digital polarimeter (JASCO Corporation, Tokyo, Japan). UV spectra were recorded on a Beckman DU 640 spectrophotometer (Beckman Coulter Inc., Brea, CA, USA). CD spectra were measured on a JASCO J-715 spectropolarimeter (JASCO Corporation). NMR spectra were recorded on JEOL JNMECP 600 (JEOL Ltd., Tokyo, Japan) and Varian-500 (Varian Medical Systems Inc., Palo Alto, CA, USA) spectrometers using TMS as an internal standard. HRESIMS spectra were measured on a Micromass EI-4000 Autospec-Ultima-TOF (Micromass Communication, Inc., Manchester, UK). Semipreparative HPLC was performed using an YMC-Pack ODS-A column (5 μm, 10 × 250 mm, YMC Co., Ltd., Kyoto, Japan). TLC and column chromatography (CC) were performed on plates precoated with silica gel GF254 (10–40 µm) and over silica gel (200–300 mesh, Qingdao Marine Chemical Factory, Qingdao, China), respectively. Size-exclusion chromatography was performed using Sephadex LH-20 (GE Healthcare, Uppsala, Sweden). The seawater was collected from Huiquan bay, Yellow Sea, China.

### 3.2. Fungal Material

The fungal strain *Aspergillus aureolatus* HDN14-107 was isolated from an unidentified sponge collected at Xisha Islands, China and was identified by ITS sequence. The ITS1-5.8S-ITS2 rDNA sequence of the fungus HDN14-107 has been submitted to GenBank with the accession number KC589122. A voucher specimen is deposited in our laboratory at −20 °C. The working strain was prepared on potato dextrose agar slants and stored at 4 °C.

### 3.3. Fermentation and Extraction

The fungus HDN14-107 was cultured under static conditions at 28 °C in 1 L Erlenmeyer flasks containing 300 mL liquid culture medium, composed of glucose (20.0 g/L), poly peptone (5.0 g/L), yeast extract (3.0 g/L), malt extract (3.0 g/L), and naturally-collected seawater (Huiquan bay, Yellow Sea, China), then adjusting the pH to 7.0. After 3 weeks of cultivation, 70 L of whole broth was filtered through cheesecloth to separate the supernatant from the mycelia. The former was extracted three times with EtOAc, while the latter was extracted three times with acetone and concentrated under reduced pressure to afford an aqueous solution, which was extracted three times with EtOAc. Both EtOAc solutions were combined and concentrated under reduced pressure to give the organic extract (20.0 g).

### 3.4. Purification

The organic extract was subjected to vacuum liquid chromatography over C_18_ ODS column using a gradient elution with H_2_O-MeOH to give four fractions (fraction 1-fraction 4). Fraction 1 was subjected to Sephadex LH-20 (GE Healthcare, Uppsala, Sweden) column chromatography eluting with CH_2_Cl_2_-MeOH (1:1), and then purified by a semi-preparative RPHPLC column (55:45 MeOH-H_2_O, 4 mL/min, YMC Co., Ltd.) to provide compound **3** (43 mg, *t*_R_ 9.4 min). Fraction 2 was chromatographed by RPMPLC eluting with MeOH-H_2_O (40%~60%, 110 min, 8 mL/min) to afford fraction 2.1 and fraction 2.2. Fraction 2.1 was further separated by Sephadex LH-20 chromatograph eluting with CH_2_Cl_2_-MeOH (1:1) and then on a semi-preparative RPHPLC column (60:40 MeOH-H_2_O, 4 mL/min) to provide compound **10** (5 mg, *t*_R_ 9.6 min). Fraction 2.2 was further purified by Sephadex LH-20 chromatograph eluting with CH_2_Cl_2_-MeOH (1:1), and then purified by semi-preparative RPHPLC column (57:43 MeOH-H_2_O, 4 mL/min) to provide compound **2** (5 mg, *t*_R_ 14.7 min) and **8** (7 mg, *t*_R_ 12 min). Fraction 3 was chromatographed by RPMPLC eluting with MeOH-H_2_O (40%~100%, 165 min, 8 mL/min) and further purified by Sephadex LH-20 chromatograph eluting with MeOH to afford fraction 3.1, fraction 3.2 and fraction 3.3. Fraction 3.1 was rechromatographed by semi-preparative RPHPLC (60:40 MeOH-H_2_O, 3 mL/min) to afford compound **11** (35.9 mg, *t*_R_ 16.0 min), compound **9** (10 mg, *t*_R_ 18 min), compound **6** and **7** (50 mg, *t*_R_ 20 min). Fraction 3.2 was further purified by semi-preparative RPHPLC (60:40 MeOH-H_2_O, 3 mL/min) to afford compound **1** (3 mg, *t*_R_ 22.4 min). Fraction 3.3 was purified by semi-preparative RPHPLC (70:30 MeOH-H_2_O, 3 mL/min) to give compound **13** (10 mg, *t*_R_ 16.3 min), compound **12** (31.2 mg, *t*_R_ 19.8 min) and fraction 3.3.1. Fraction 3.3.1 was further purified by semi-preparative RPHPLC (65:35 MeOH-H_2_O, 3 mL/min) to afford compound **5** (10 mg, *t*_R_ 18 min). Fraction 4 was rechromatographed on semi-preparative RPMPLC eluting with MeOH-H_2_O (65%~100%, 115 min, 8 mL/min) to give fraction 4.1, and then further purified by Sephadex LH-20 chromatograph eluting with CH_2_Cl_2_-MeOH (1:1) afford fraction 4.1.1 and fraction 4.1.2. Fraction 4.1.1 was eluted with MeOH-H_2_O (65:35 MeOH-H_2_O, 3 mL/min) on semi-preparative RPHPLC to provide compound **14** (5.3 mg, *t*_R_ 24.7 min). Fraction 4.1.2 was purified by semi-preparative RPHPLC (45:55 CH_3_CN-H_2_O, 30 min, 3 mL/min) to provide compound **4** (9.6 mg, *t*_R_ 32 min).

Austalide S (**1**): white amorphous powder (MeOH); [*α*]${}_{D}^{20}$ = −22 (*c* 0.15, CHCl_3_); UV (MeOH) *λ*_max_ (log *ε*) 218 (4.32), 265 (3.40); ECD (7.0 × 10^−4^ M, MeCN) *λ*_max_ (Δ*ε*) 299 nm (+0.53), 229 nm (−1.53), 209 nm (+4.09); ^1^H NMR and ^13^C NMR data see Table 1 and Table 2; HRESIMS *m*/*z* 401.2323 [M + H]^+^ (calcd for C_2__4_H_33_O_5_, 401.2323).

Austalide T (**2**): white amorphous powder (MeOH); [*α*]${}_{D}^{20}$ = −88 (*c* 0.07, CHCl_3_); UV (MeOH) *λ*_max_ (log *ε*) 221 (3.61), 265 (3.52); ECD (5.9 × 10^−4^ M, MeCN) *λ*_max_ (Δ*ε*) 269 nm (−2.91), 229 nm (+2.45), 212 nm (−2.99); ^1^H NMR and ^13^C NMR data see Table 1 and Table 2; HRESIMS *m*/*z* 503.2276 [M + H]^+^ (calcd for C_27_H_35_O_9_, 503.2276).

Austalide U (**3**): white amorphous powder (MeOH); [*α*]${}_{D}^{20}$ = −46 (*c* 0.13, CH_2_Cl_2_); UV (MeOH) *λ*_max_ (log *ε*) 221 (4.23), 266 (3.91); ECD (6.5 × 10^−4^ M, MeCN) *λ*_max_ (Δ*ε*) 270 nm (−4.65), 229 nm (+4.53), 212 nm (−4.78); ^1^H NMR and ^13^C NMR data see Table 1 and Table 2; HRESIMS *m*/*z* 461.2163 [M + H]^+^ (calcd. for C_25_H_33_O_8_, 461.2170).

Austalide A (**4**): white amorphous powder (MeOH); ECD (9.4 × 10^−4^ M, MeCN) *λ*_max_ (Δ*ε*) 264 nm (−4.83), 228 nm (+3.22), 210 nm (−2.56).

Austalide J (**9**): white amorphous powder (MeOH), ECD (1.3 × 10^−3^ M, MeCN) *λ*_max_ (Δ*ε*) 265 nm (−2.69), 229 nm (+3.15), 210 nm (−1.73).

13-*O*-deacethylaustalide I (**10**): white amorphous powder (MeOH); ECD (4.5 × 10^−4^ M, MeCN) *λ*_max_ (Δ*ε*) 268 nm (−1.82), 229 nm (+1.24), 213 nm (−2.54).

### 3.5. Computation Section

Conformational searches were run employing the “systematic” procedure implemented in Spartan’14 [16], using MMFF (Merck molecular force field). All MMFF minima were reoptimized with DFT calculations at the B3LYP/6-31+G(d) level using the Gaussian 09 program [17]. The geometry was optimized starting from various initial conformations, with vibrational frequency calculations confirming the presence of minima. Time-dependent DFT calculations were performed on three lowest-energy conformations (Supplementary Figure S27) for the configuration using 20 excited states, and using a polarizable continuum model (PCM) for acetonitrile. ECD spectra were generated using the program SpecDis [18] by applying a Gaussian band shape with 0.20 eV width, from dipole-length rotational strengths. The dipole velocity forms yielded negligible differences.

## 4. Conclusions

In summary, fourteen austalides meroterpenoids, including three new ones, were isolated from sponge-derived fungus *Aspergillus aureolatus* HDN14-107. All the chemical structures, including absolute configurations, were established. Compound **1** is the first case of austalides with the terpene ring fused to the chroman ring in *trans* configuration. This study has revealed a new carbon skeleton, which might be useful for further study of antiviral mechanisms and developing antiviral activity against influenza virus A (H1N1).

## Figures and Tables

**Table 1:** ^1^H (500 MHz) NMR Data for **1**–**3**.

Position	1 ^a^	2 ^a^	3 ^b^
1	5.18, s	5.12, s	5.16, s
12	1.93, d (10.8);	2.56, d (16.2);	2.30, dd (15.9, 2.1);
	1.67, m	2.17, d (17.9)	2.18, dd (15.6, 4.4)
13	1.70, m	5.10, brs	4.08, m
14	0.90, d (12.2)		
17	3.08, m		
18	1.51, m	1.11, m	1.87, m; 1.60, m
19	1.99, d (12.6);	1.94, m	1.91, m
	0.68, t (13.3)		
21	1.59, d (7.6)	2.43, d (8.0)	2.46, dd (5.8, 3.2)
22	2.45, d (15.4)	2.87, dd (18.8, 8.4)	2.91, d (6.3)
	3.05, d (15.4)	2.97, d (18.8)	
23	2.05, s	1.99, s	2.05, s
24	1.34, s	1.23, s	1.24, s
25	0.96, s	1.54, s	1.40, s
26	0.78, s	1.32, s	1.54, s
27	0.99, s	1.00, s	0.97, s
28		4.13, s	4.03, s
30		2.06, s	
5-OH	7.84, s		

^a^ Spectrum was recorded in CDCl_3_; ^b^ spectrum was recorded in CD_3_CN.

**Table 2:** ^13^C (125 MHz) NMR Data for **1**–**3**.

Position	1 ^a^	2 ^a^	3 ^b^
1	69.9, CH_2_	68.2, CH_2_	68.2, CH_2_
3	173.8, C	169.4, C	169.0, C
4	101.7, C	113.9, C	107.6, C
5	152.2, C	155.4, C	155.0, C
6	115.0, C	115.6, C	116.6, C
7	161.4, C	157.9, C	157.9, C
8	110.9, C	107.4, C	114.6, C
9	143.9, C	145.6, C	146.0, C
11	77.2, C	75.6, C	77.0, C
12	43.6, CH_2_	38.0, CH_2_	41.8, CH_2_
13	20.1, CH_2_	70.9, CH	69.1, CH
14	54.9, CH	86.2, C	86.6, C
15	38.8, C	85.0, C	84.5, C
17	78.5, CH	117.8, C	171.0, C
18	26.9, CH_2_	29.7, CH_2_	30.1, CH_2_
19	35.6, CH_2_	31.0, CH_2_	31.2, CH_2_
20	38.9, C	40.2, C	39.4, C
21	59.1, CH	36.0, CH	36.1, CH
22	17.6, CH_2_	18.0, CH_2_	17.9, CH_2_
23	11.3, CH_3_	10.6, CH_3_	9.9, CH_3_
24	24.7, CH_3_	27.5, CH_3_	27.0, CH_3_
25	28.1, CH_3_	25.5, CH_3_	28.7, CH_3_
26	15.4, CH_3_	29.4, CH_3_	25.2, CH_3_
27	15.0, CH_3_	18.0, CH_3_	18.2, CH_3_
28		62.0, CH_3_	61.3, CH_3_
29		169.5, C	
30		21.2, CH_3_	

^a^ Spectrum was recorded in CDCl_3_; ^b^ spectrum was recorded in CD_3_CN.

## Supplementary Materials

The materials are available online at www.mdpi.com/1660-3397/14/7/131/s1. Computational data, HPLC analysis of the fungal metabolite under different culture conditions, as well as NMR spectra for compounds **1**–**3**.
