# Supplementary material for: Austalides S-U, New Meroterpenoids from the Sponge-Derived Fungus Aspergillus aureolatus HDN14-107
Source: Mar Drugs. 2016 Jul 14;14(7):131. doi: 10.3390/md14070131 (PMC4962021; doi:10.3390/md14070131)
Supplement: Supplementary file 1 [file marinedrugs-14-00131-s001.pdf]

Supplementary Materials: Austalides S-U, New Meroterpenoids from the Sponge-Derived Fungus *Aspergillus aureolatus* HDN14-107

Jixing Peng, Xiaomin Zhang, Wei Wang, Tianjiao Zhu, Qianqun Gu and Dehai Li

HRESIMS, 1D and 2D NMR data of **1** in $CDCl_3$ (Figures S1–S9);
HRESIMS, 1D and 2D NMR data of **2** in $CDCl_3$ (Figures S10–S17);
HRESIMS, 1D and 2D NMR data of **3** in CD_3CN (Figures S17–S25);
HPLC analysis of the EtOAc extract of *Aspergillus aureolatus* HDN14-107 (Figure S26);
Computational data (Figures S27).

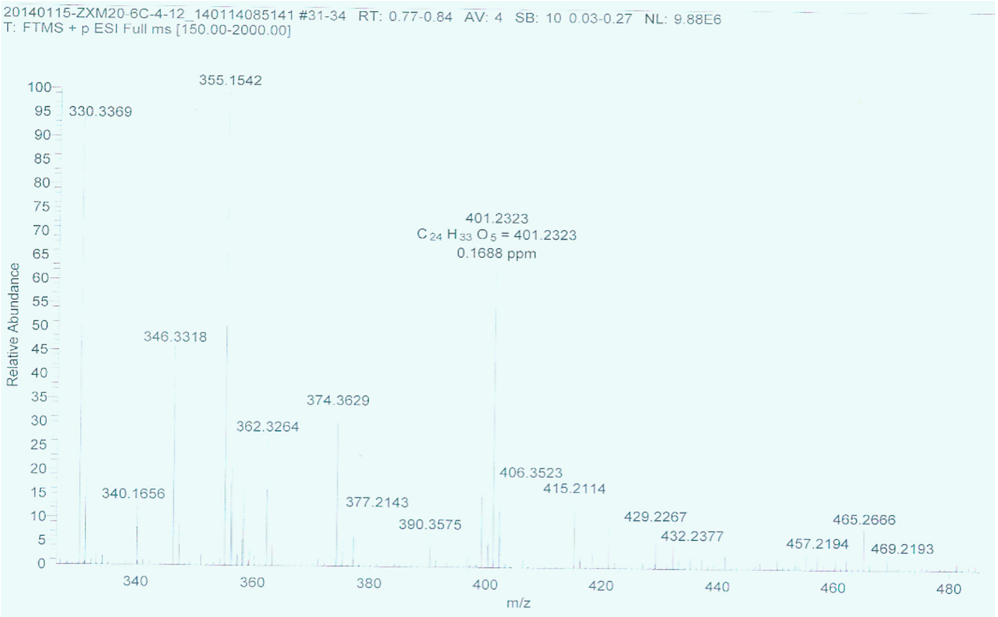

Figure S1. HR-ESI-MS of **1**.

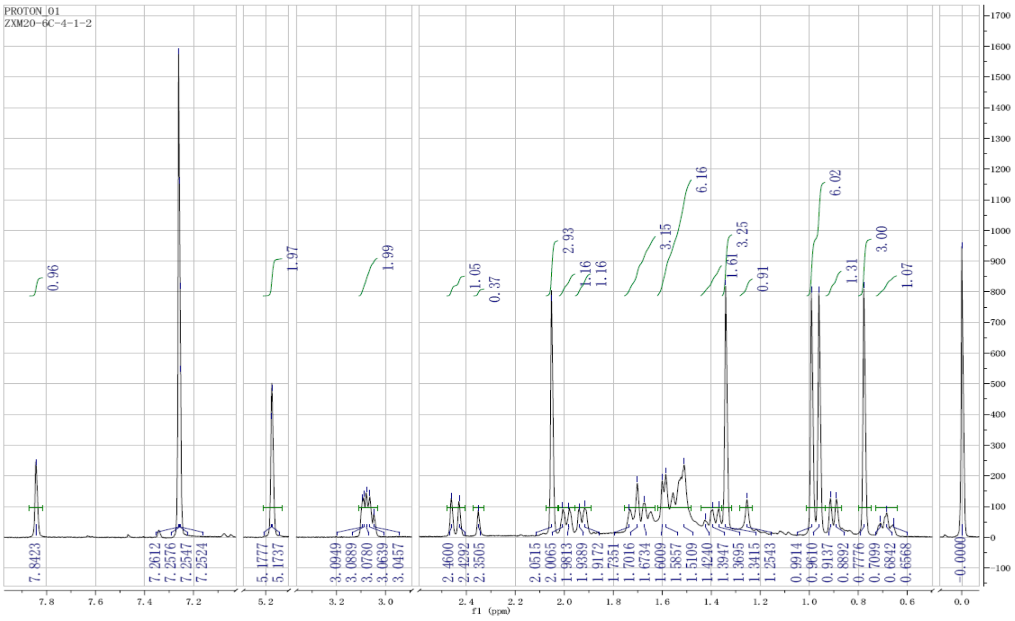

Figure S2. ^{1}H NMR (500 MHz) spectrum of **1** in $CDCl_3$.

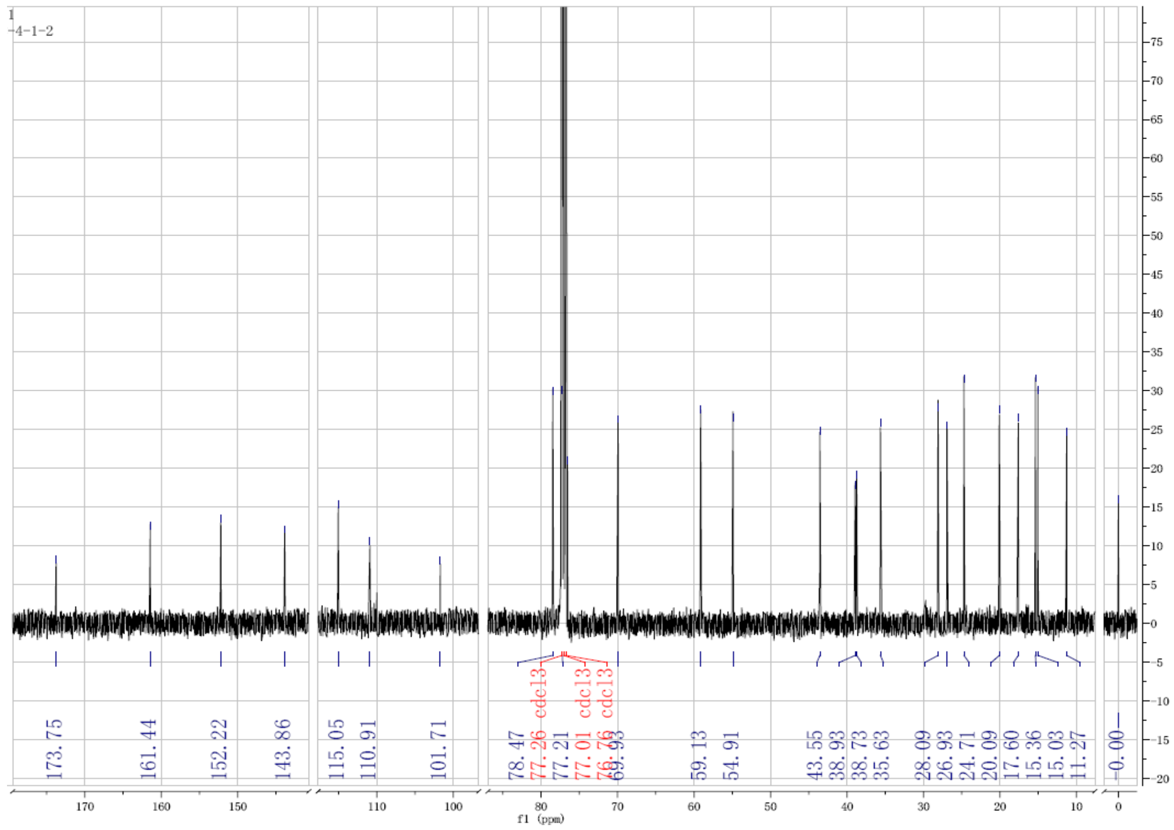

Figure S3. ^{13}C NMR spectrum of **1** in $CDCl_3$.

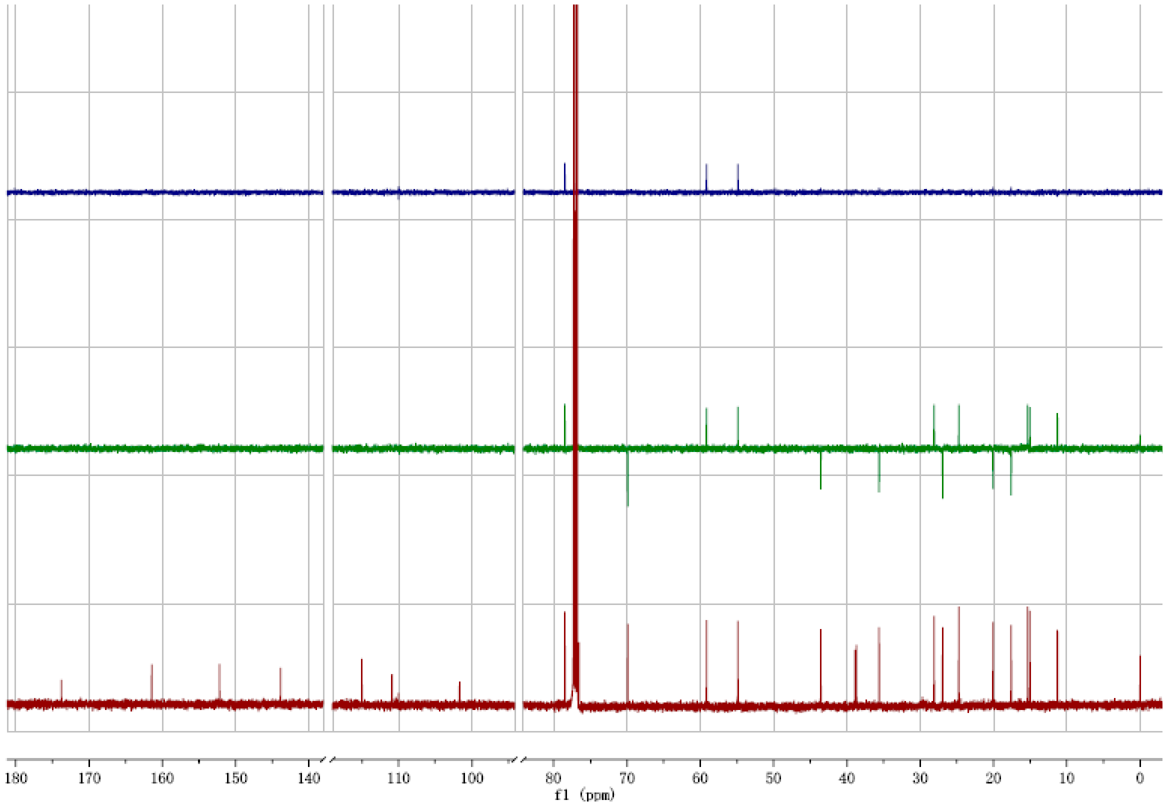

Figure S4. DEPT spectrum of **1** in $CDCl_3$.

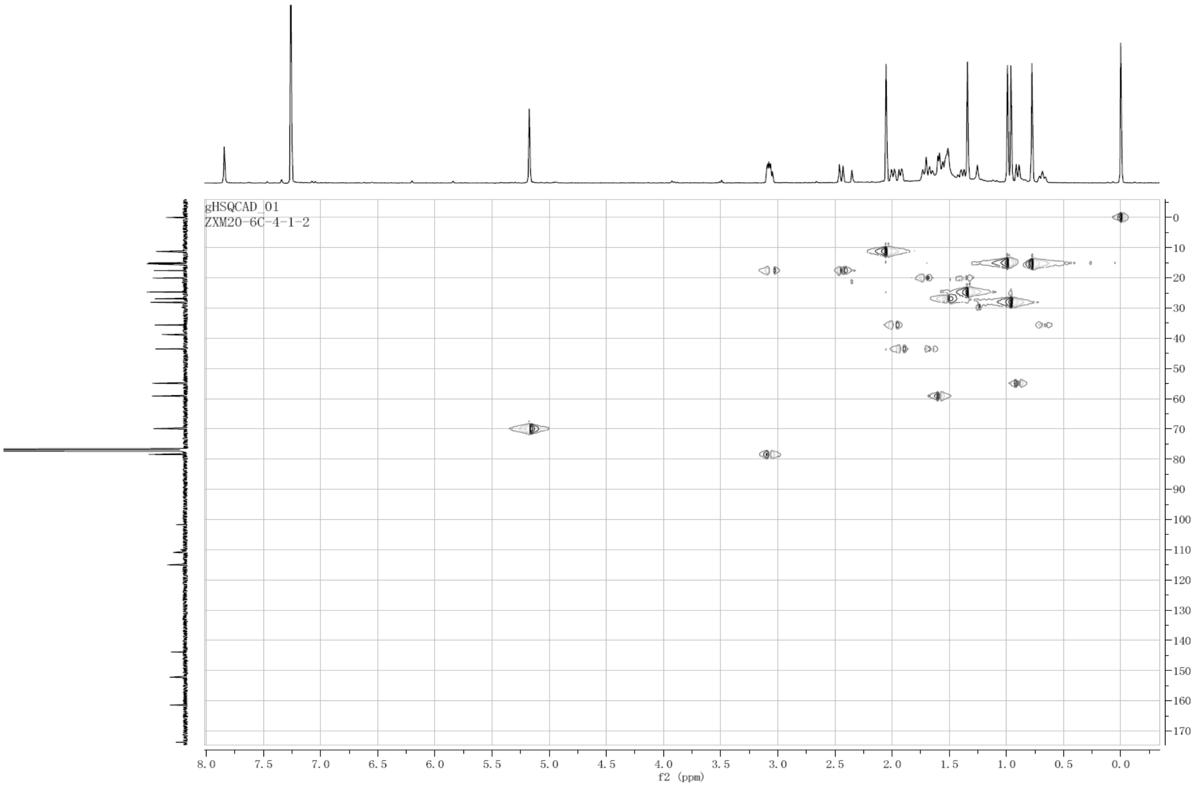

Figure S5. HMQC spectrum of **1** in $CDCl_3$.

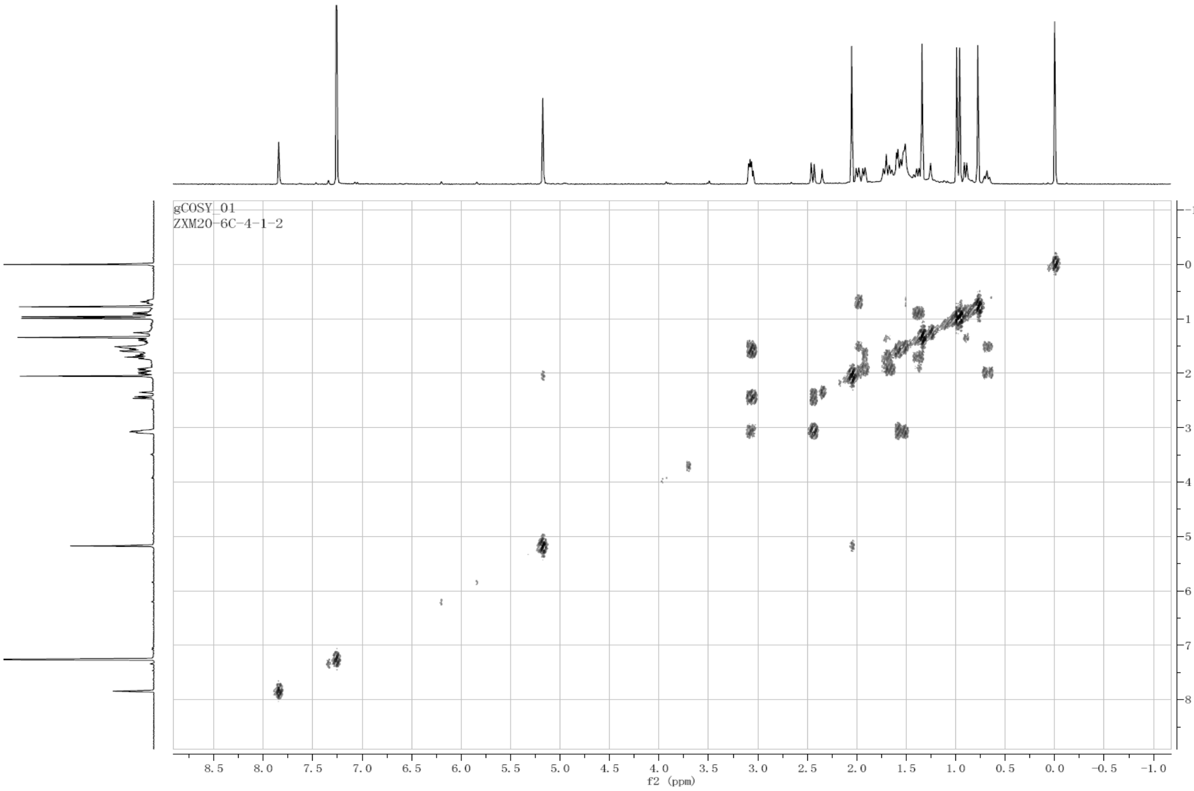

Figure S6. COSY spectrum of **1** in $CDCl_3$.

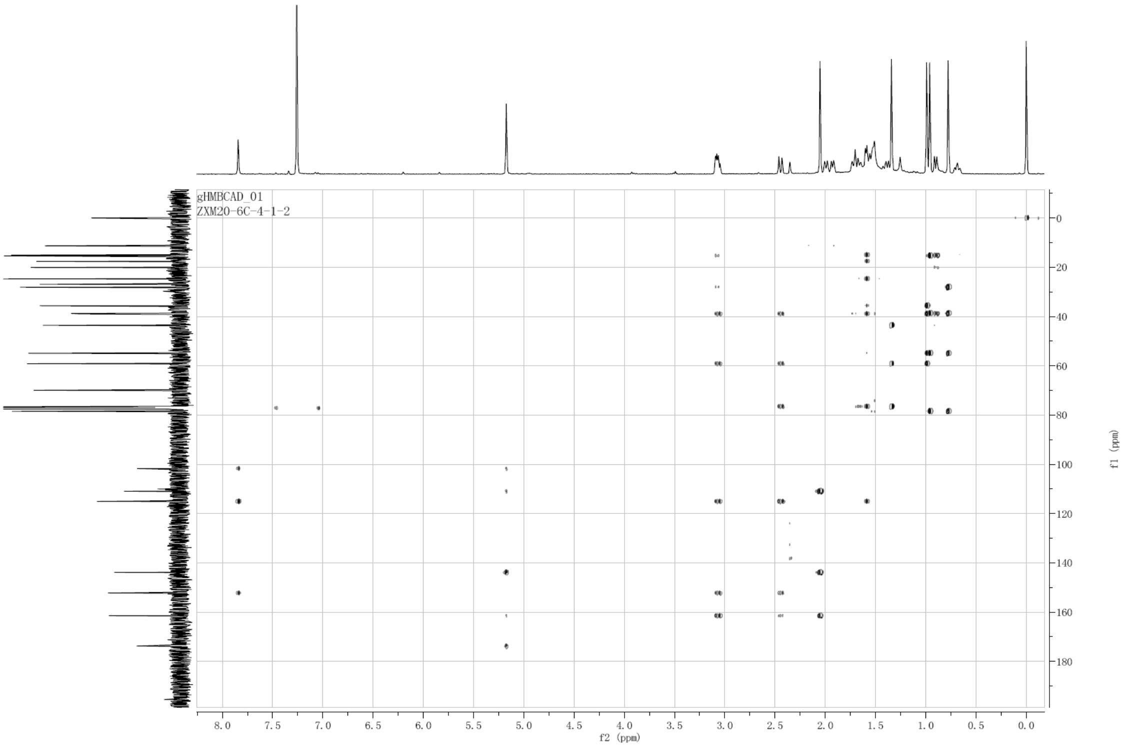

Figure S7. HMBC spectrum of **1** in $CDCl_3$.

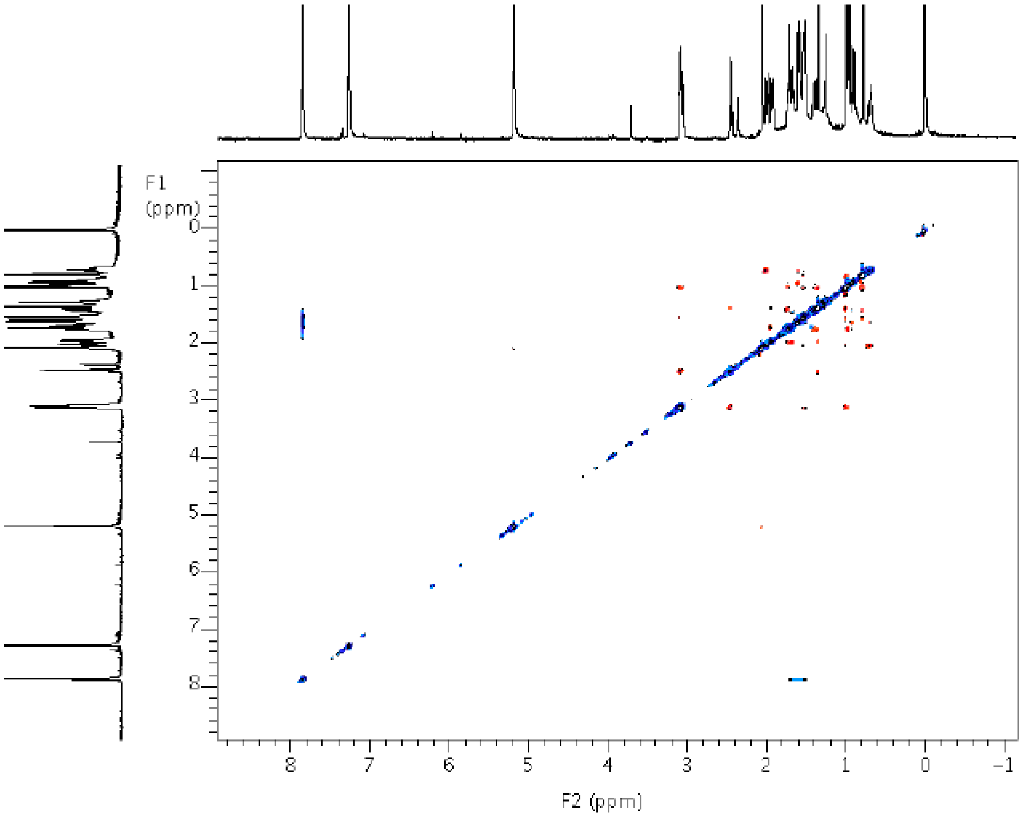

Figure S8. NOESY spectrum of **1** in $CDCl_3$.

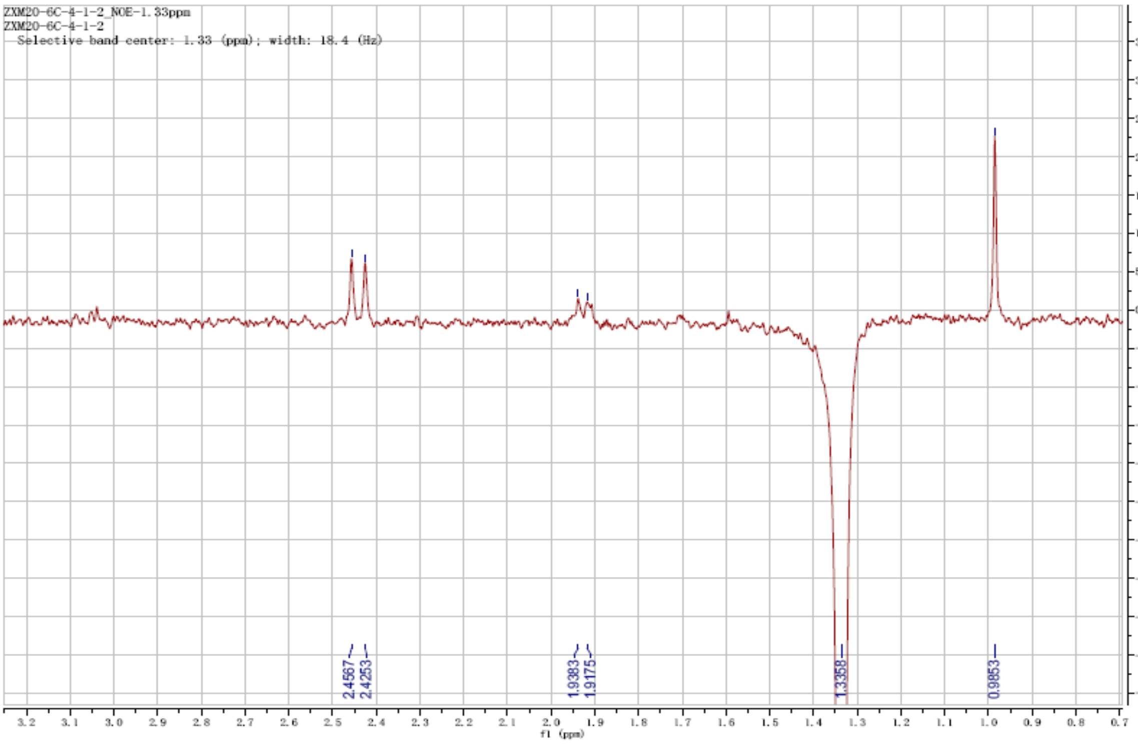

Figure S9. NOE spectrum of **1** in $CDCl_3$.

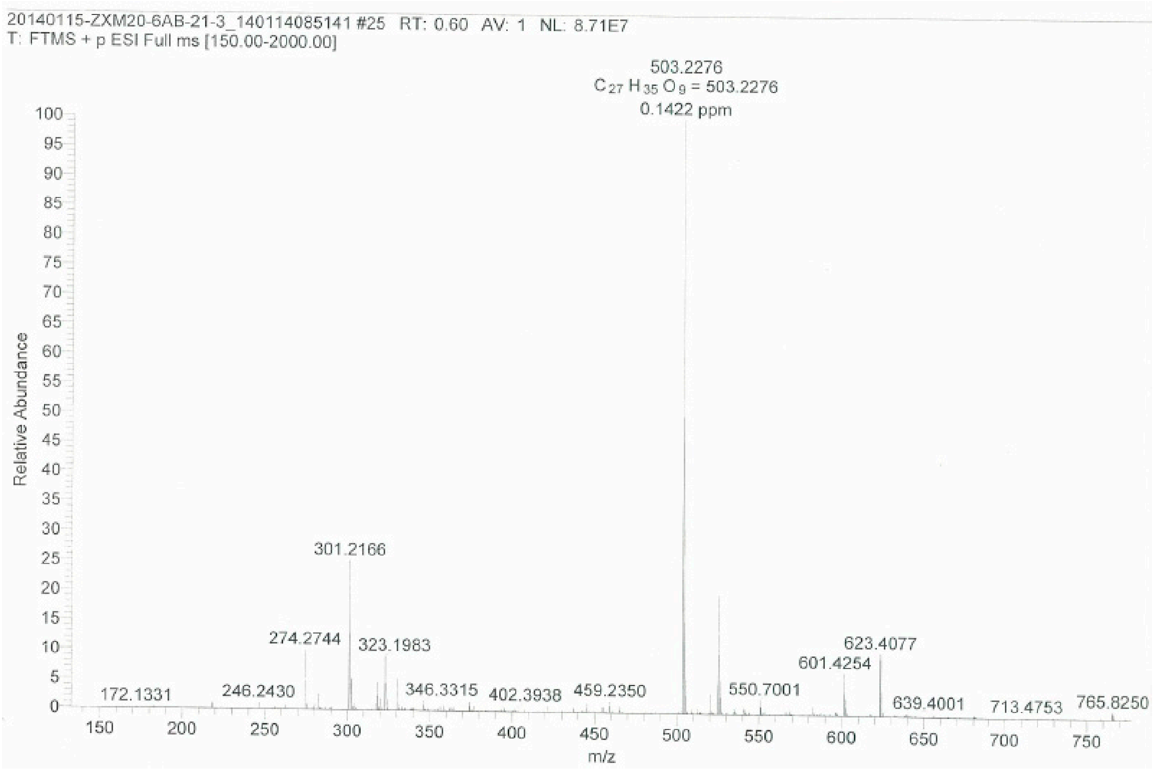

Figure S10. HR-ESI-MS of **2**.

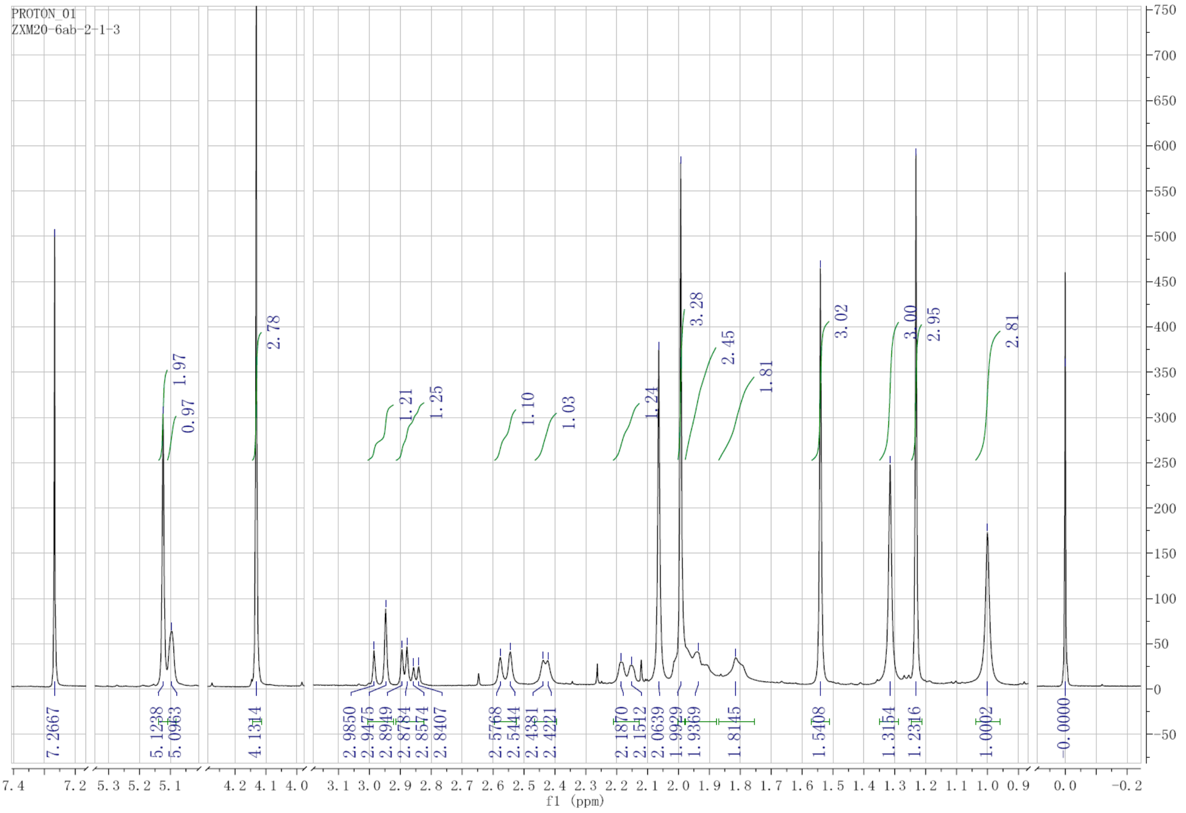

Figure S11. ^{1}H NMR (500 MHz) spectrum of **2** in $CDCl_3$.

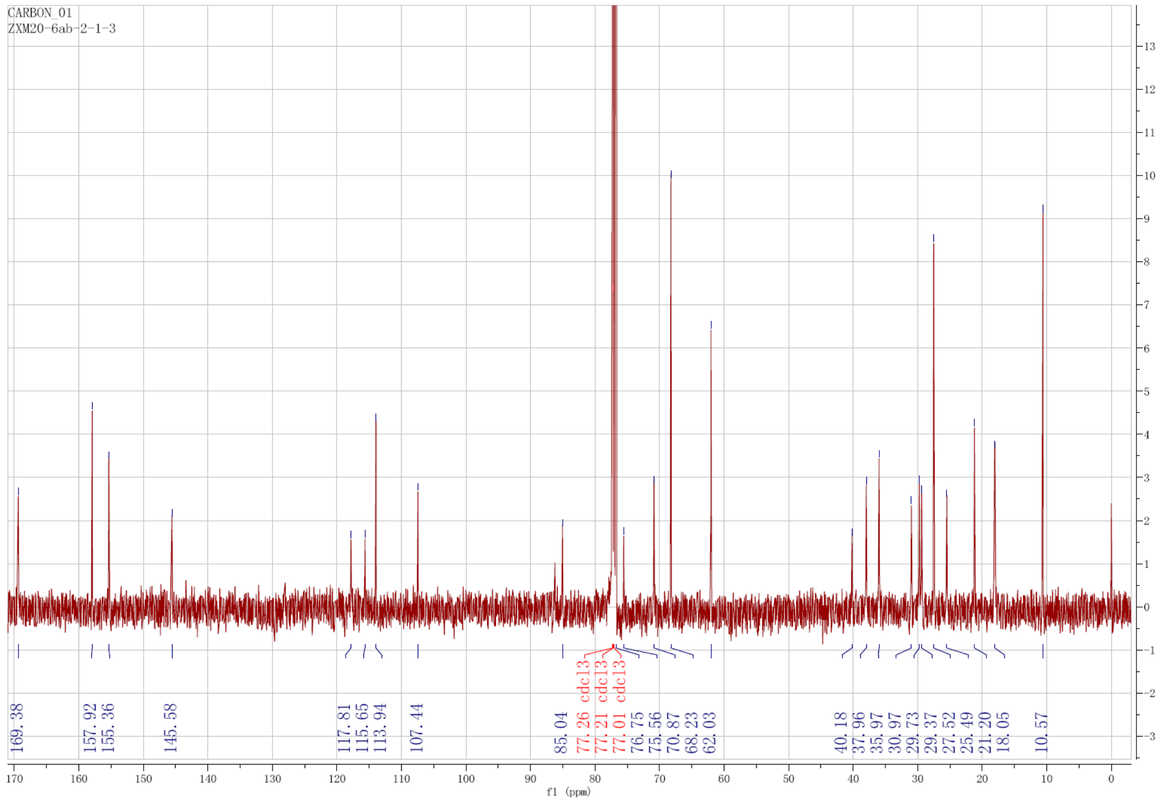

Figure S12. ^{13}C NMR spectrum of **2** in $CDCl_3$.

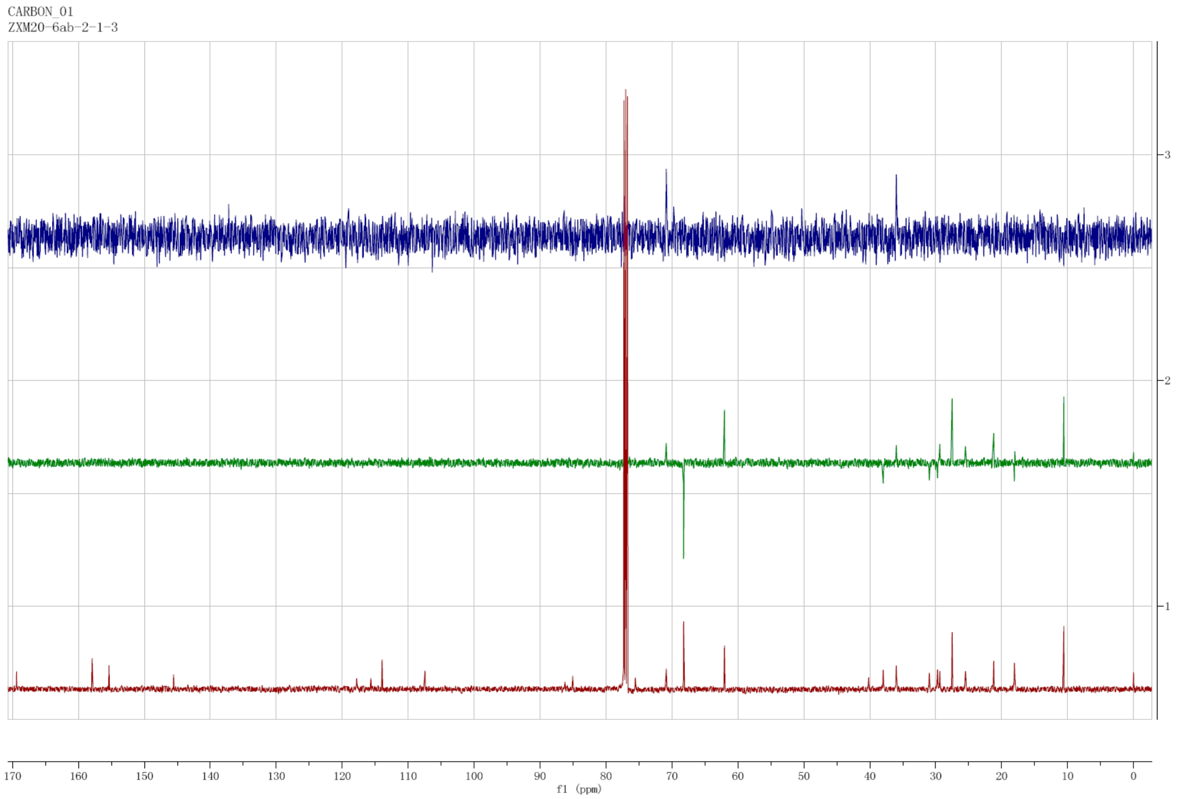

Figure S13. DEPT spectrum of **2** in $CDCl_3$.

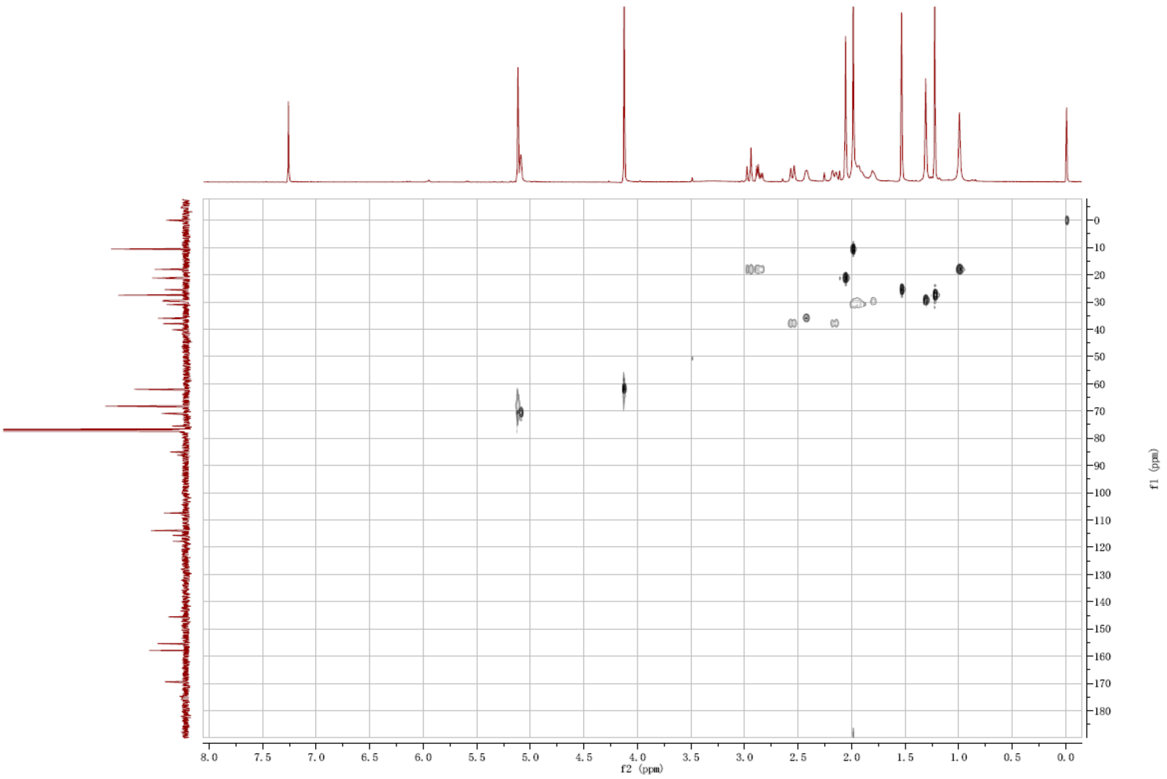

Figure S14. HMQC spectrum of **2** in $CDCl_3$.

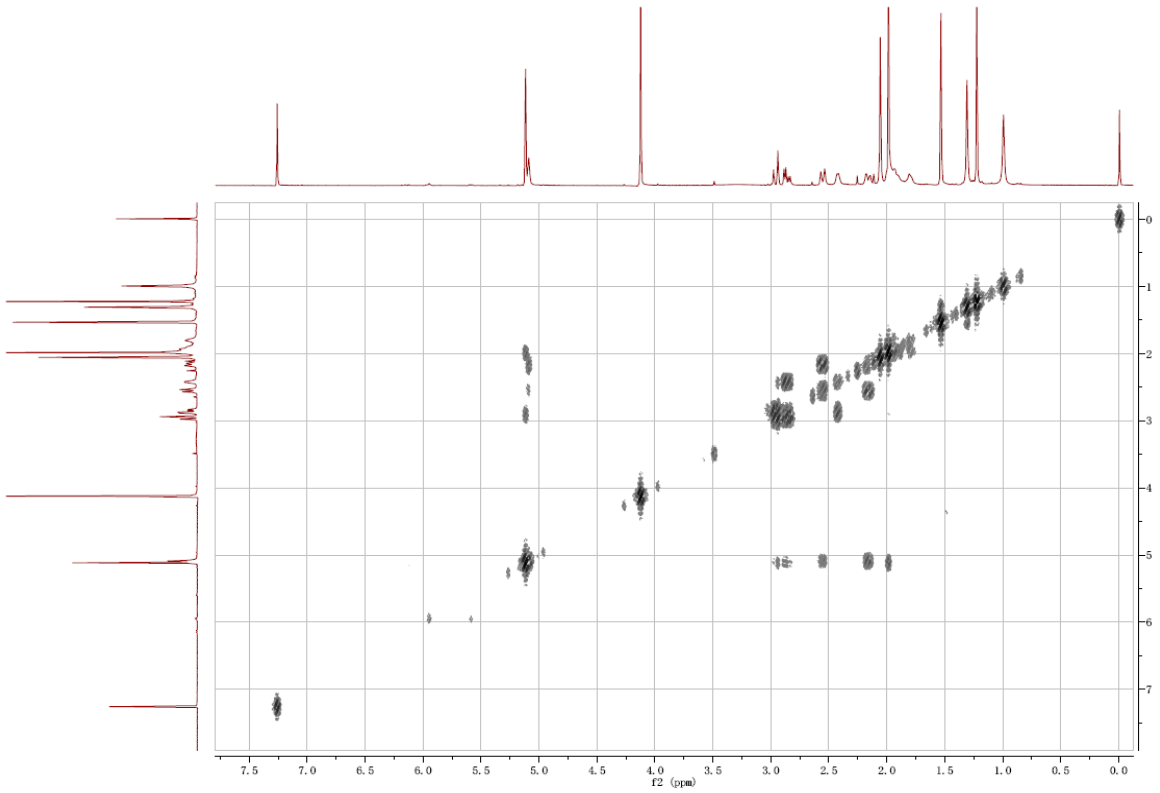

Figure S15. COSY spectrum of **2** in $CDCl_3$.

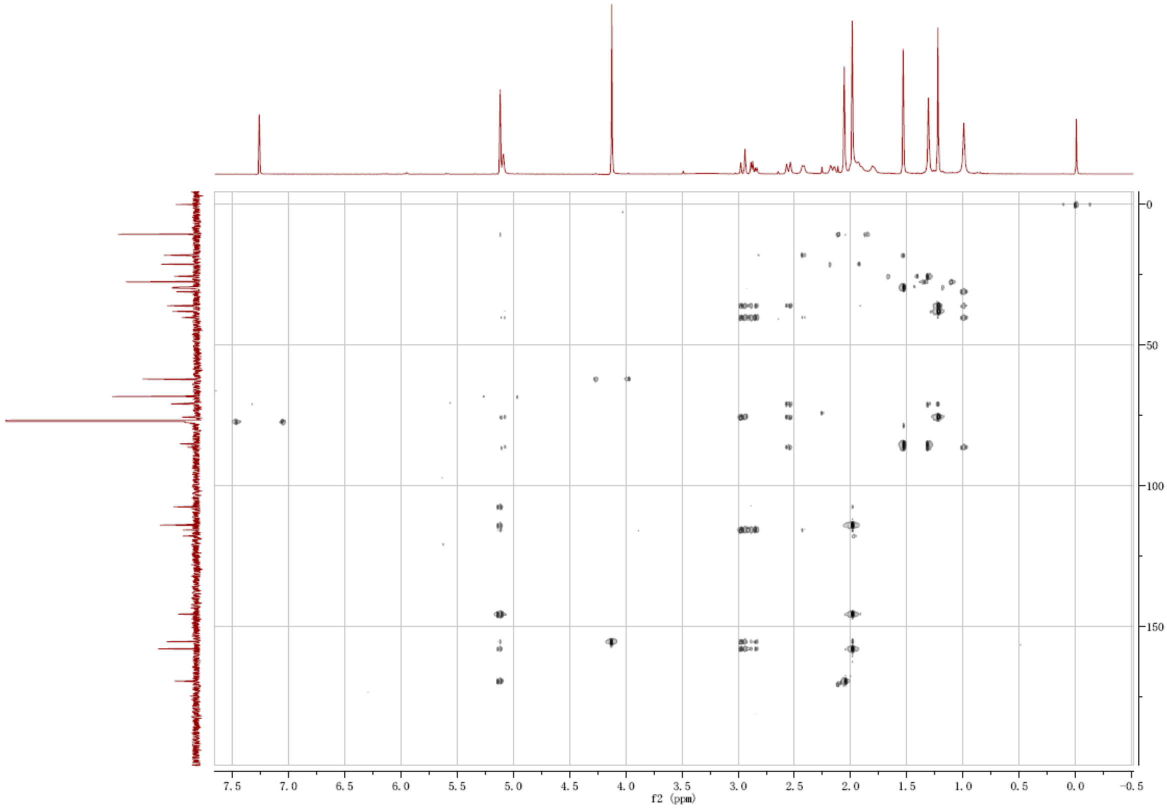

Figure S16. HMBC spectrum of **2** in $CDCl_3$.

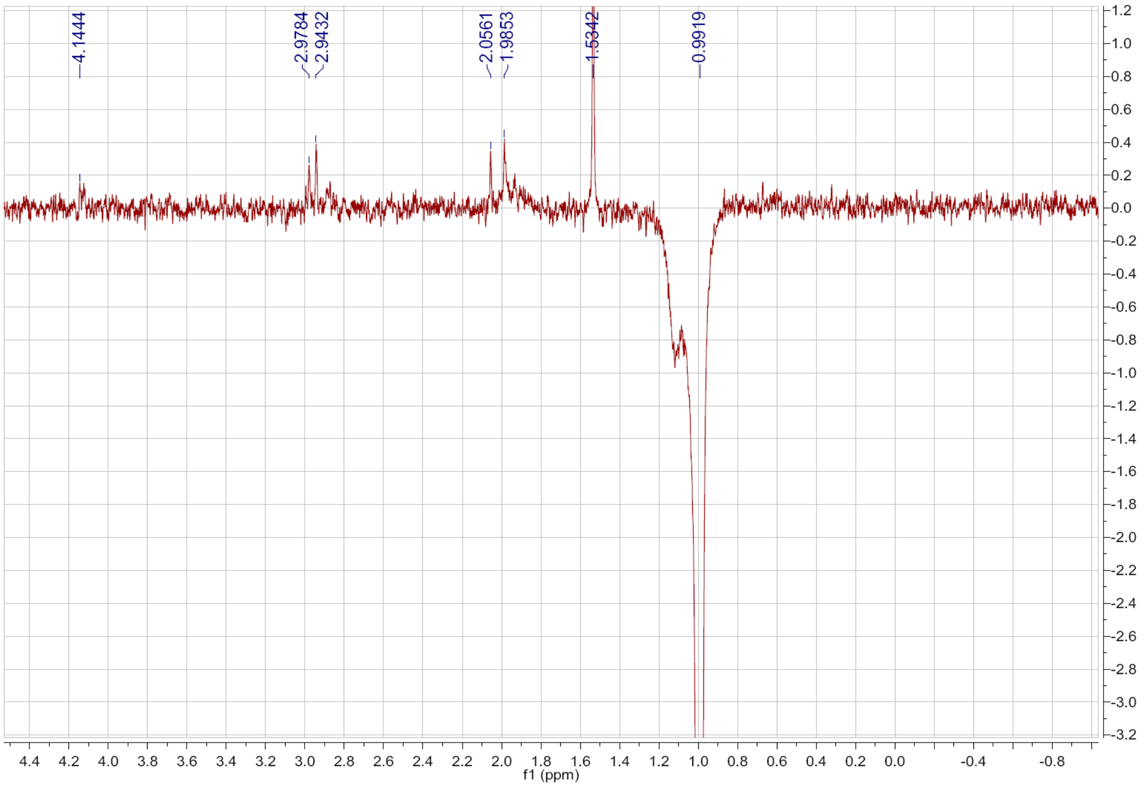
4.1444
2.9784
2.9432
2.0561
1.9853
1.5342
0.9919
f1 (ppm)

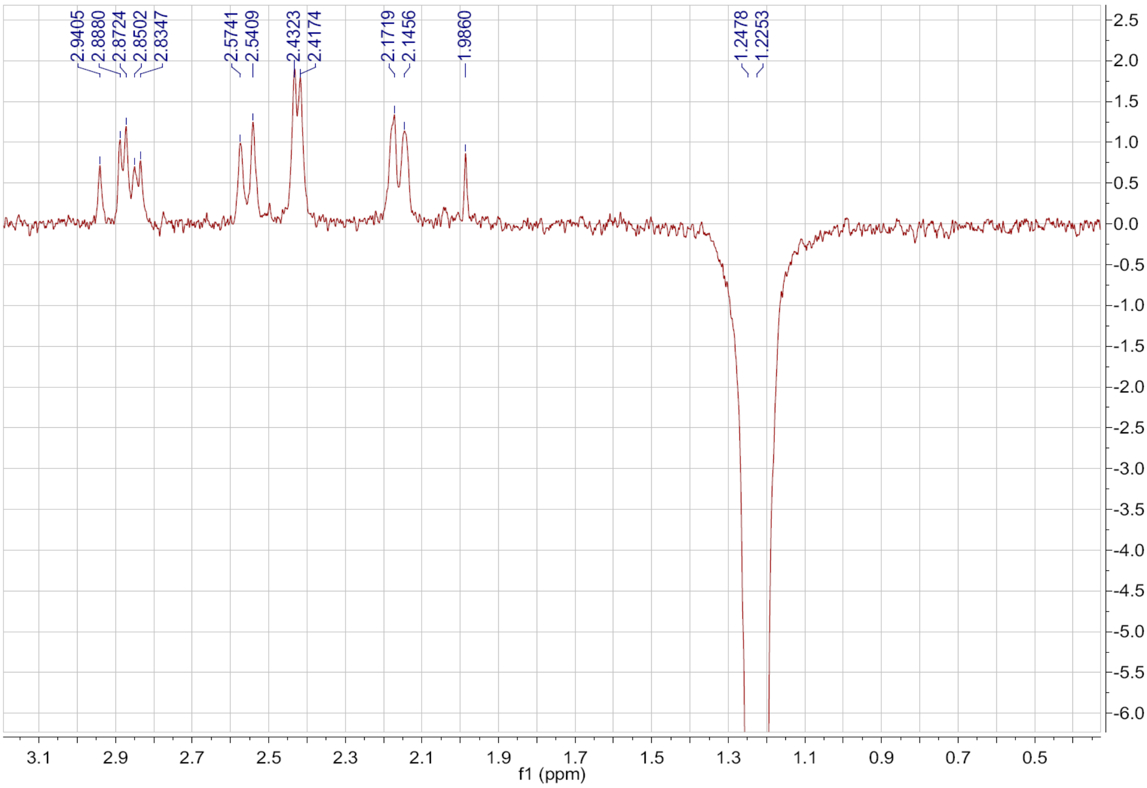
2.9405
2.8880
2.8724
2.8502
2.8347
2.5741
2.5409
2.4323
2.4174
2.1719
2.1456
1.9860
1.2478
1.2253
f1 (ppm)

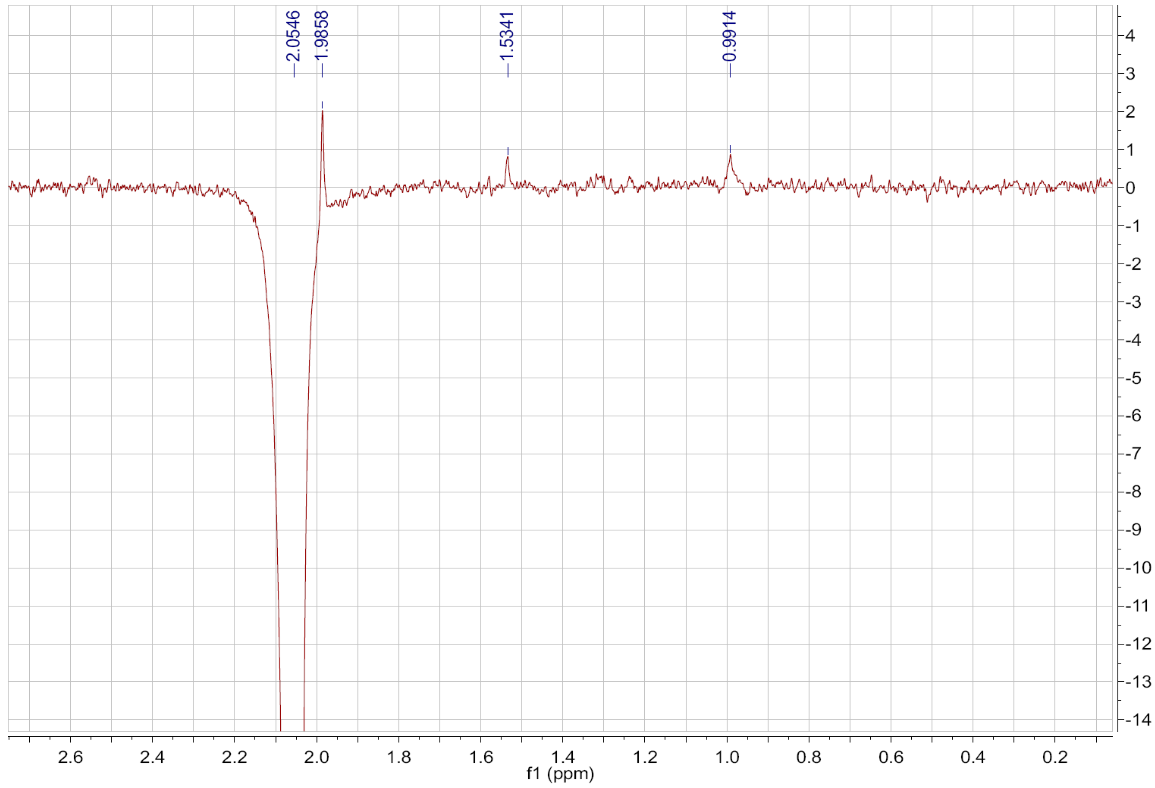

Figure S17. NOE spectra of **2** in $CDCl_3$.

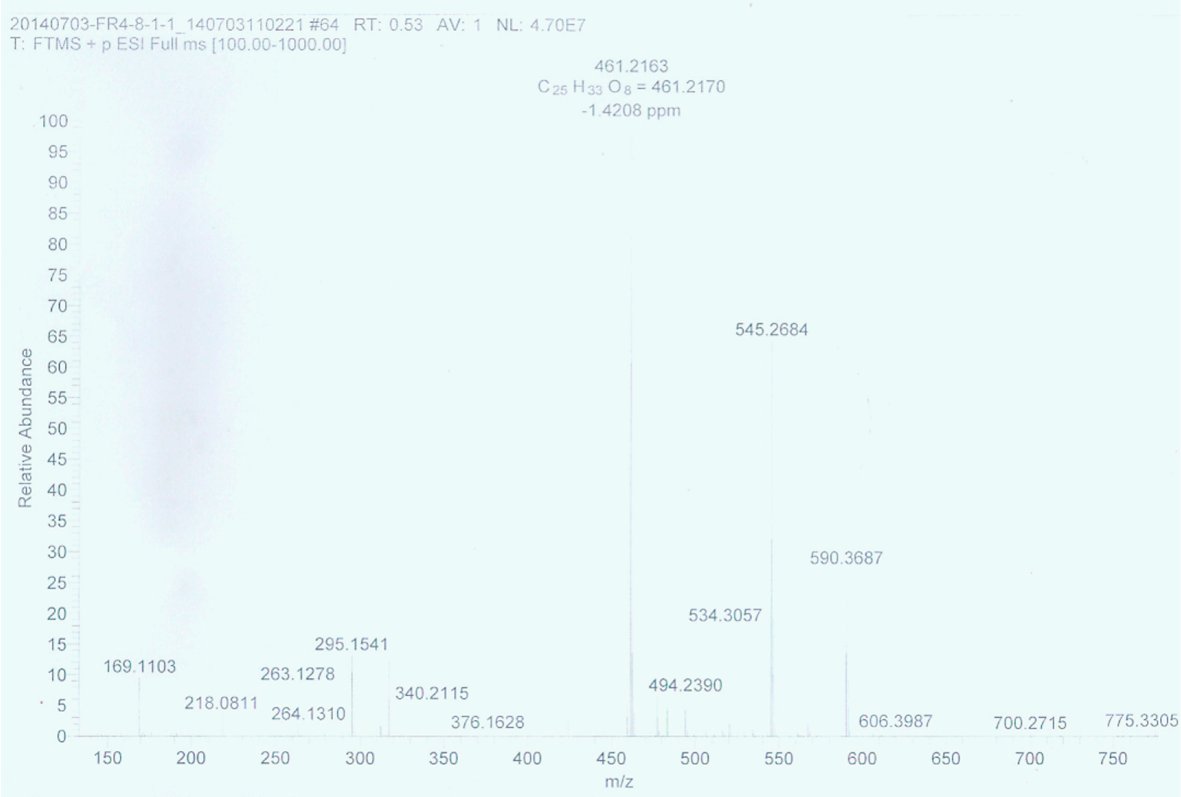

Figure S18. HRESIMS of Compound **3**.

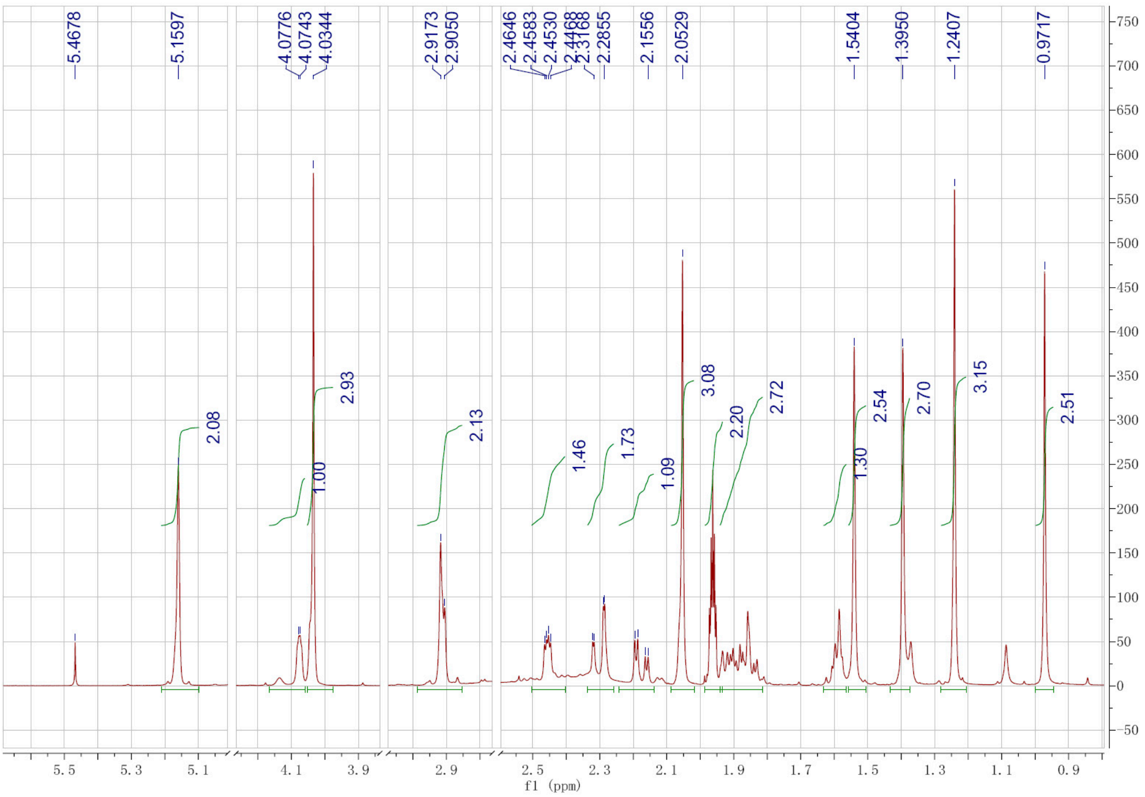

Figure S19. ^{1}H NMR (500 MHz) spectrum of Compound **3** in CD_3CN.

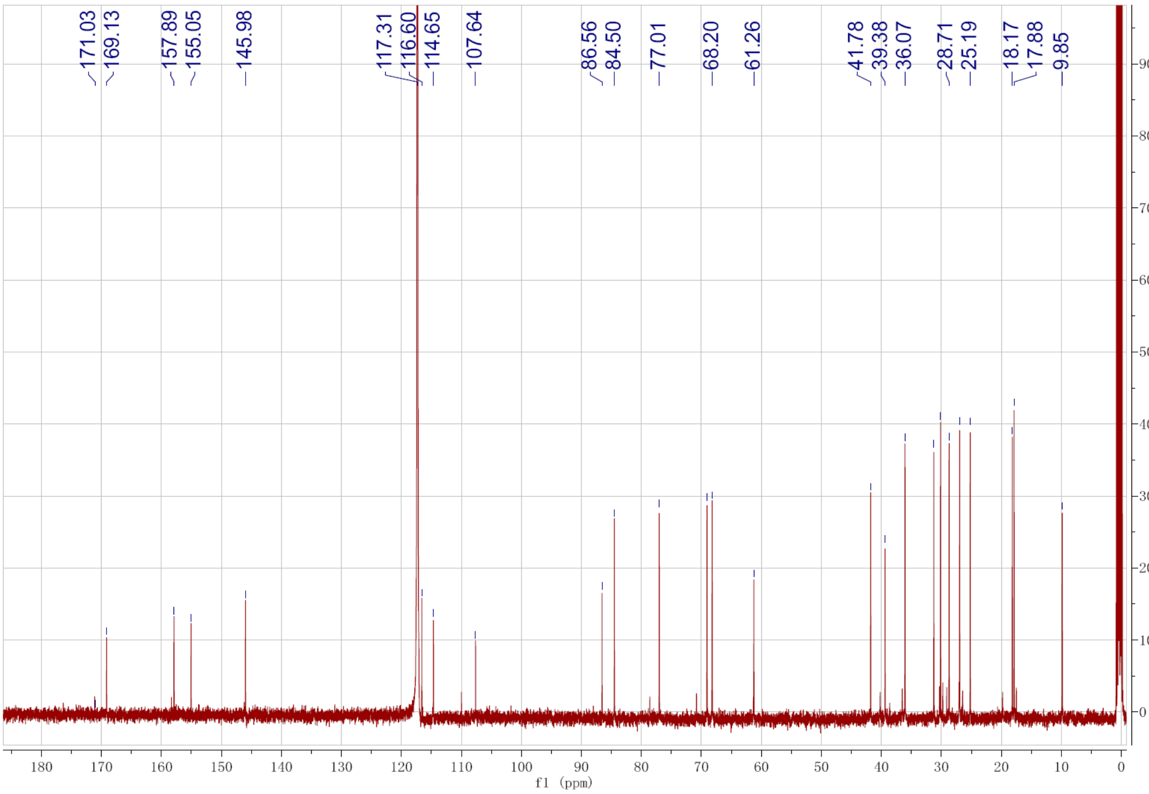

Figure S20. ^{13}C NMR spectrum of Compound **3** in CD_3CN.

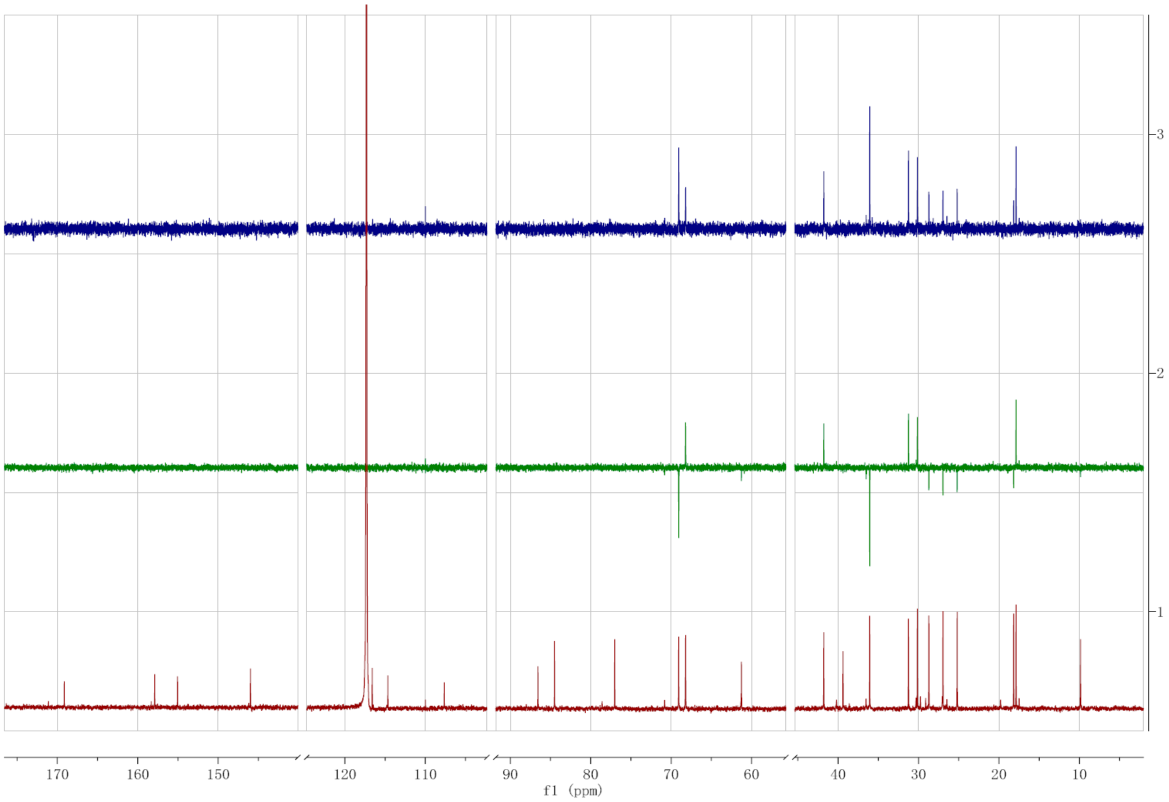

Figure S21. DEPT spectrum of Compound **3** in CD_3CN.

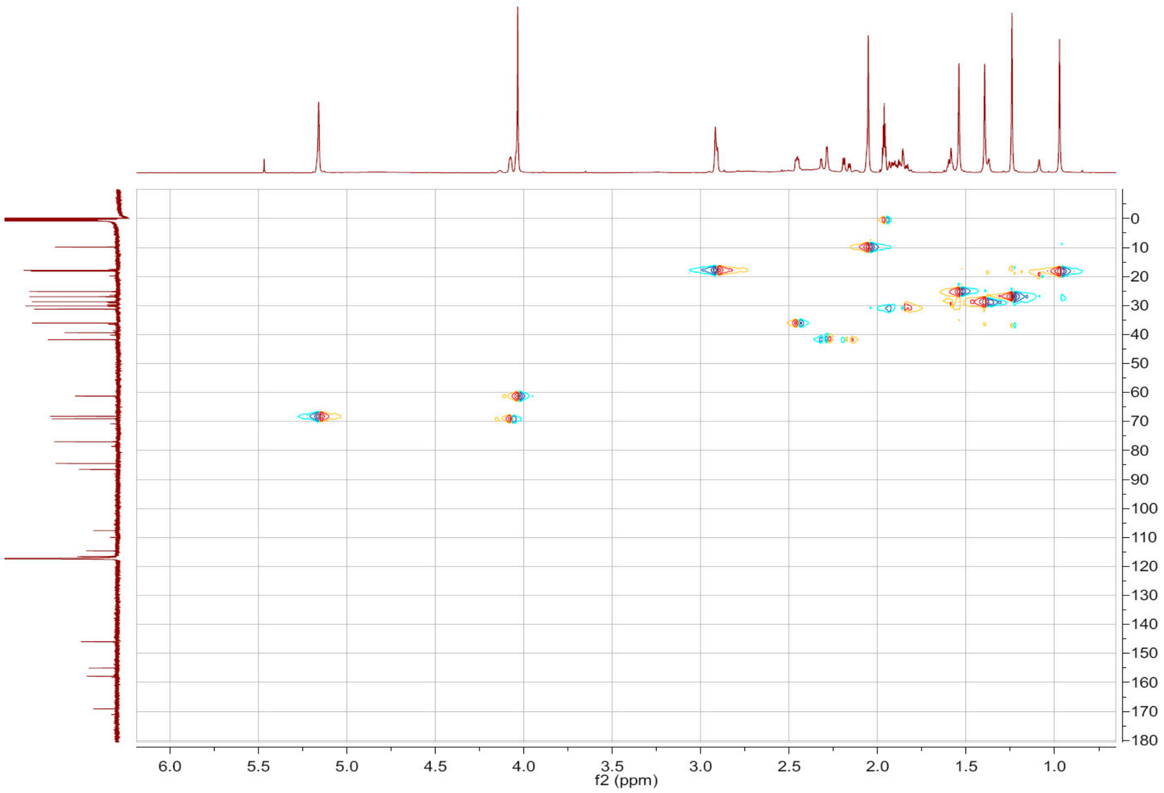

Figure S22. HMQC spectrum of Compound **3** in CD_3CN.

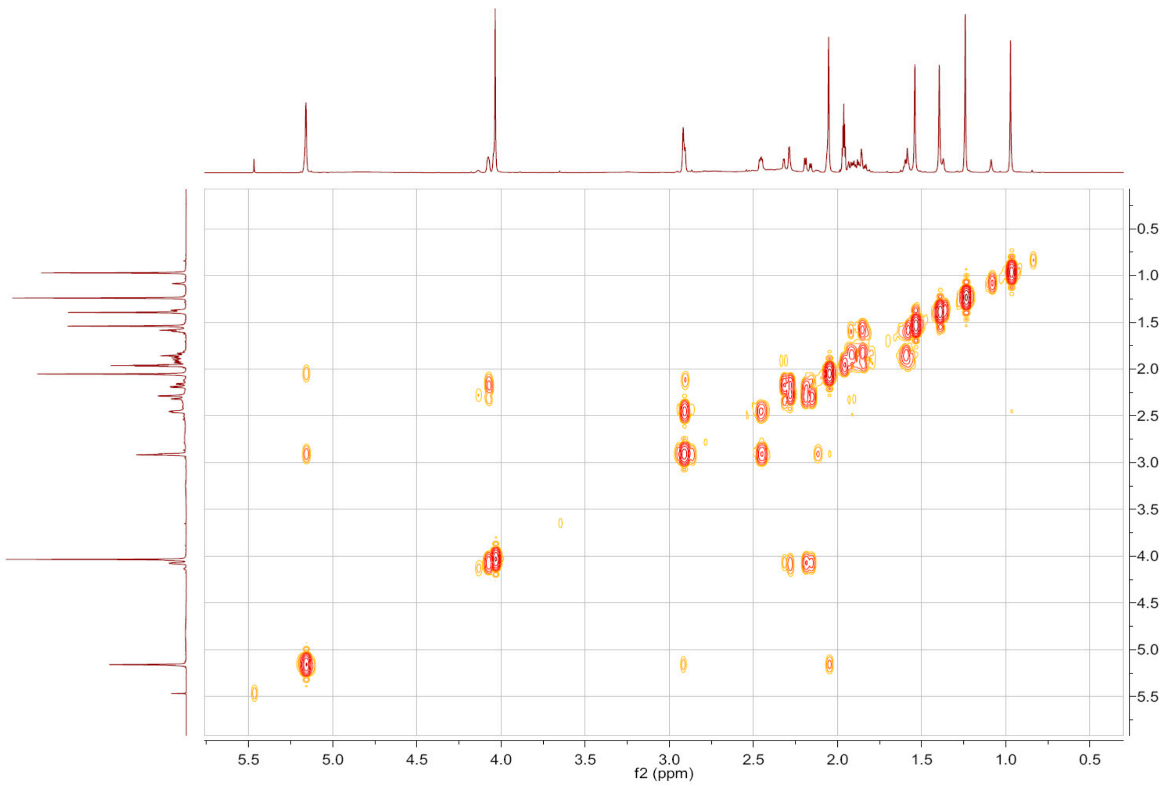

Figure S23. ^{1}H–^{1}H COSY spectrum of Compound **3** in CD_3CN.

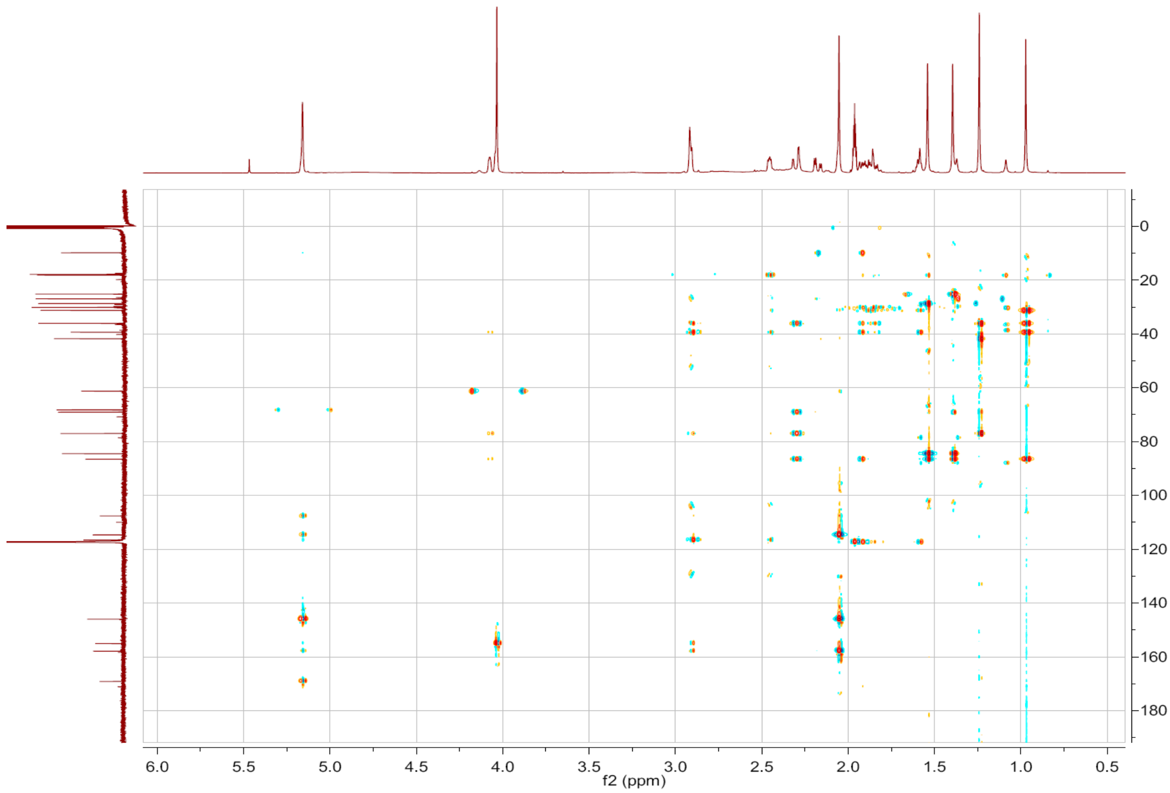

Figure S24. HMBC spectrum of Compound **3** in CD_3CN.

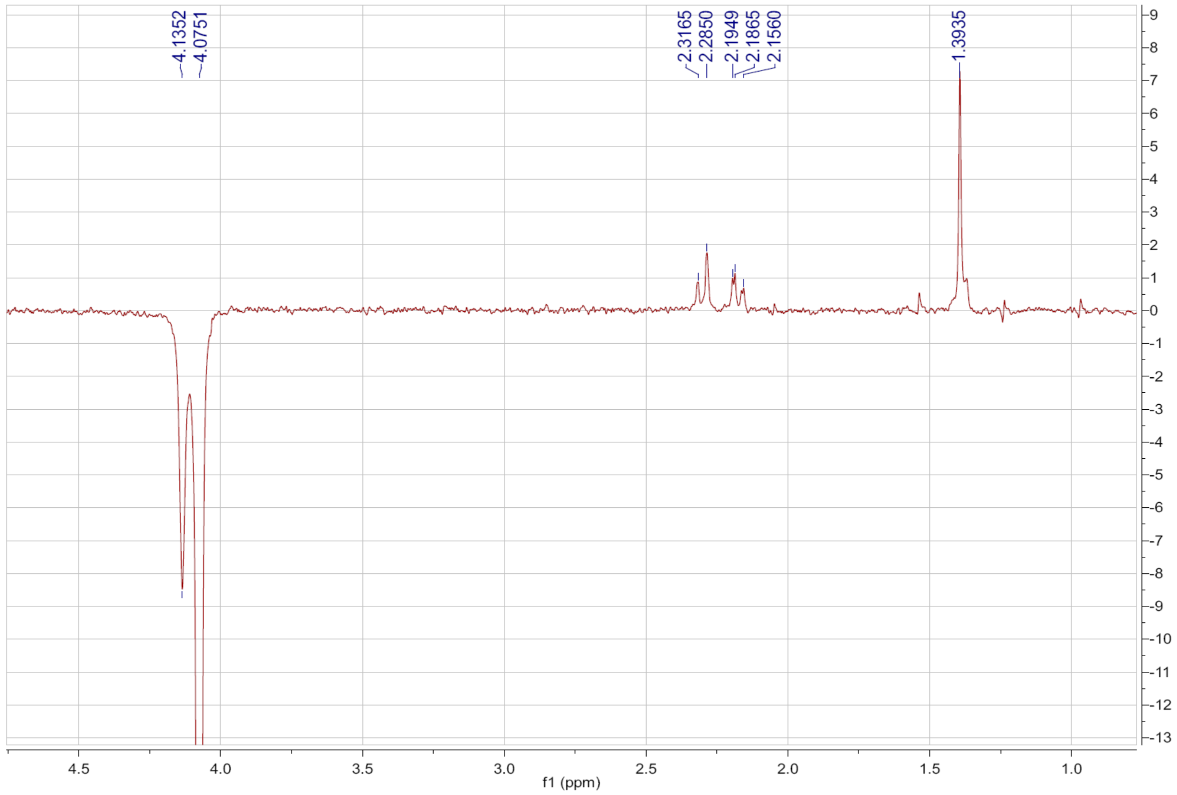
4.1352
4.0751
2.3165
2.2850
2.1949
2.1865
2.1560
1.3935
f1 (ppm)

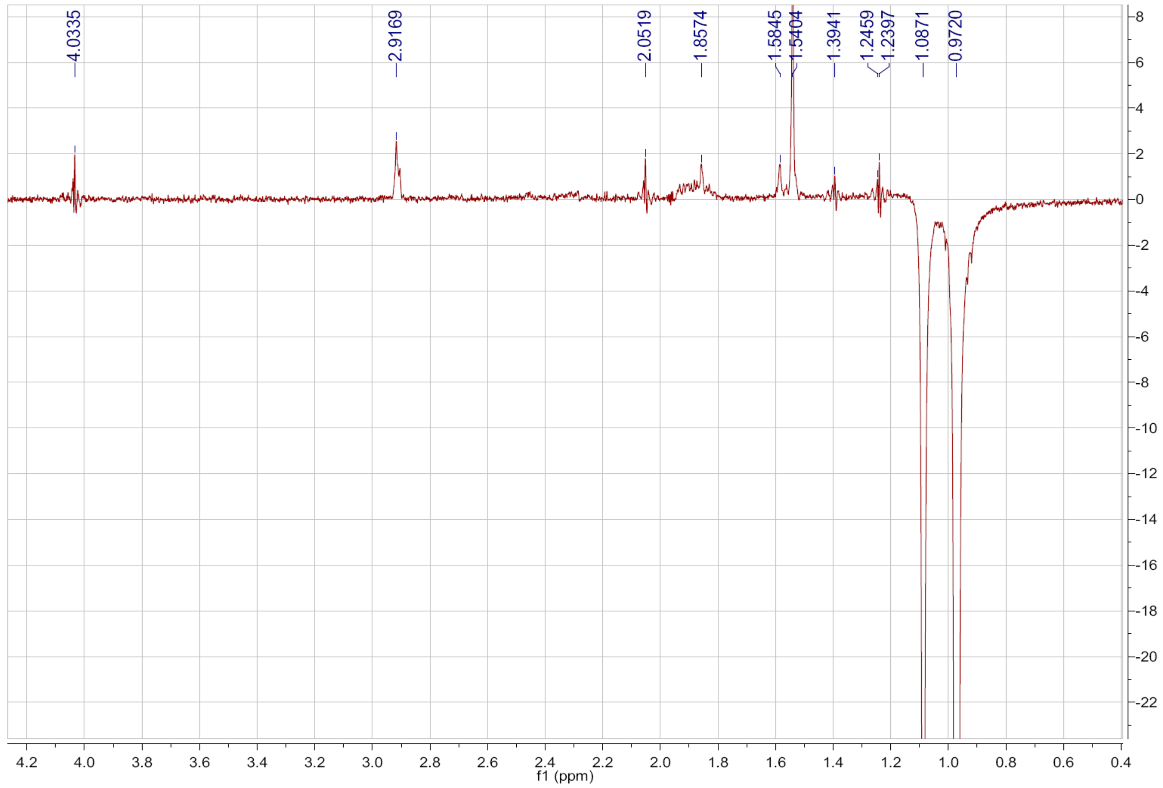
4.0335
2.9169
2.0519
1.8574
1.5845
1.5404
1.3941
1.2459
1.2397
1.0871
0.9720
f1 (ppm)

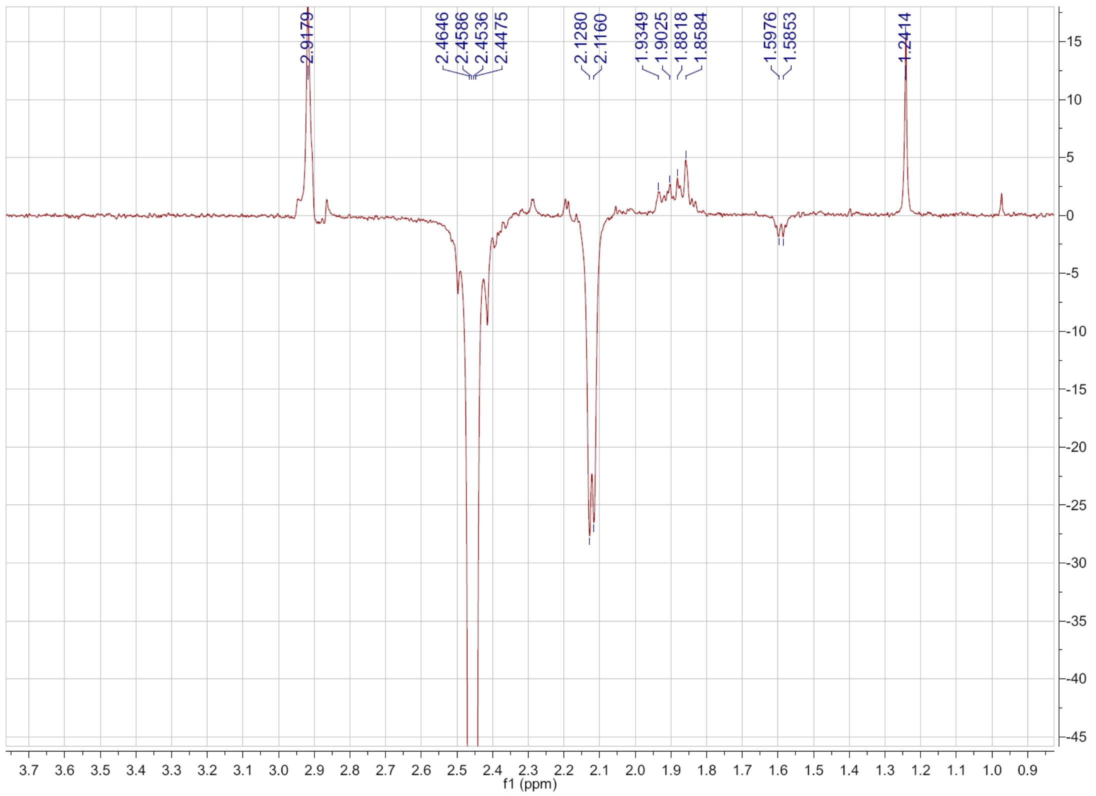

Figure S25. NOE spectra of Compound **3** in CD_3CN.

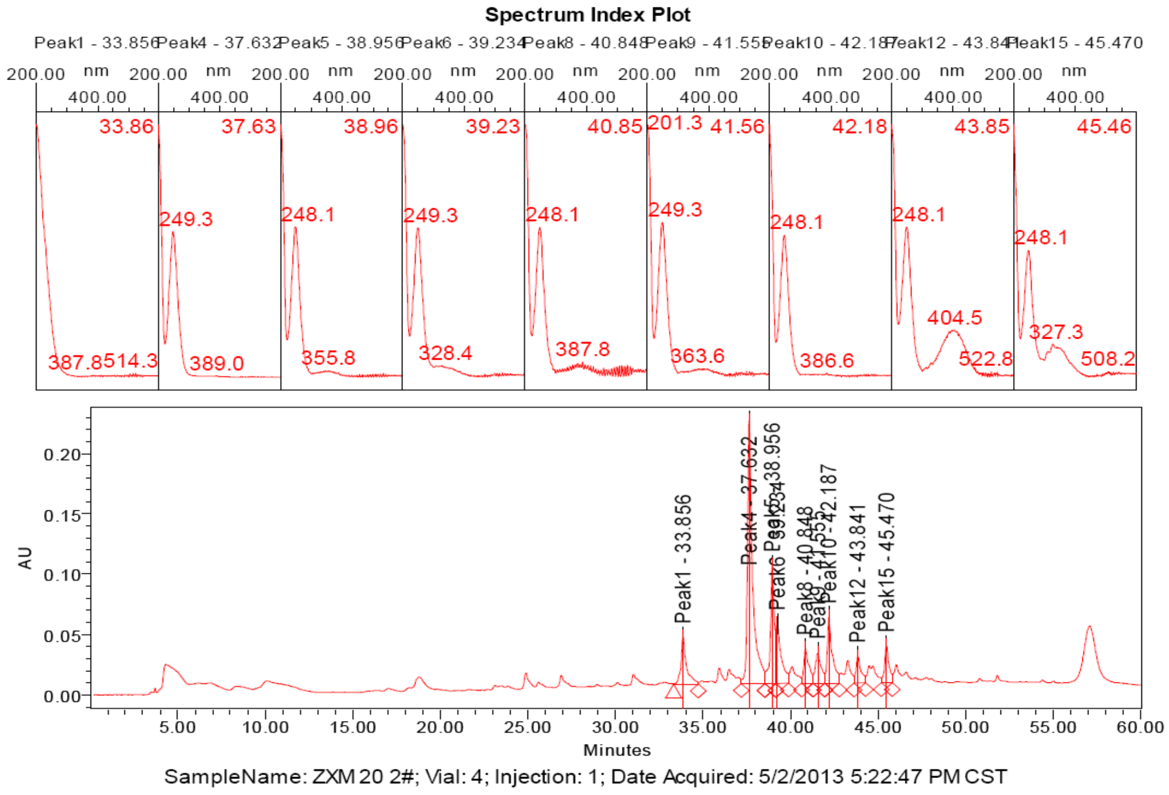

Figure S26. HPLC analysis of the EtOAc extract of *Aspergillus aureolatus* HDN14-107.

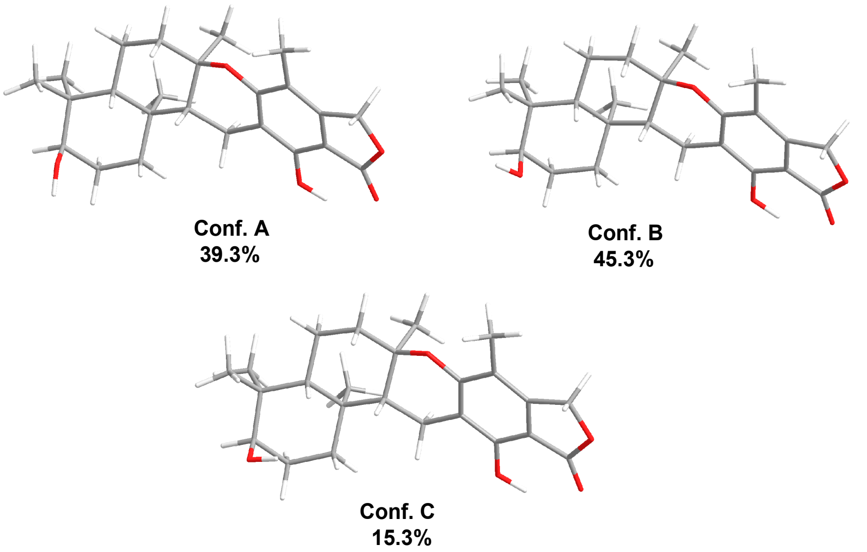

Figure S27. DFT-optimized structures for low-energy conformers of (11*R*, 14*R*, 17*R*, 20*S*, 21*R*)-**1** at B3LYP/6-31+G (d) level in acetonitrile (Conformer populations were calculated using the Gibbs free energy and Boltzmann population at 298 K estimated thereof).
